# Use of Boundary-Driven Nonequilibrium Molecular Dynamics for Determining Transport Diffusivities of Multicomponent Mixtures in Nanoporous Materials

**DOI:** 10.1021/acs.jpcb.1c09159

**Published:** 2022-02-01

**Authors:** Maziar Fayaz-Torshizi, Weilun Xu, Joseph R. Vella, Bennett D. Marshall, Peter I. Ravikovitch, Erich A. Müller

**Affiliations:** † Department of Chemical Engineering, Imperial College London, London SW7 2AZ, United Kingdom; ‡ ExxonMobil Research and Engineering Company, Irving, Texas 75039-2298, United States; § ExxonMobil Research and Engineering Company, Annandale, New Jersey 08801, United States

## Abstract

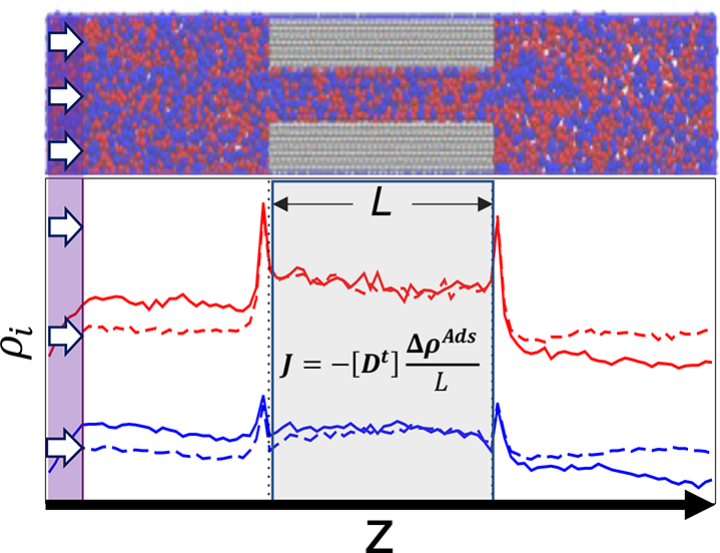

The boundary-driven molecular modeling strategy to evaluate mass transport coefficients of fluids in nanoconfined media is revisited and expanded to multicomponent mixtures. The method requires setting up a simulation with bulk fluid reservoirs upstream and downstream of a porous media. A fluid flow is induced by applying an external force at the periodic boundary between the upstream and downstream reservoirs. The relationship between the resulting flow and the density gradient of the adsorbed fluid at the entrance/exit of the porous media provides for a direct path for the calculation of the transport diffusivities. It is shown how the transport diffusivities found this way relate to the collective, Onsager, and self-diffusion coefficients, typically used in other contexts to describe fluid transport in porous media. Examples are provided by calculating the diffusion coefficients of a Lennard-Jones (LJ) fluid and mixtures of differently sized LJ particles in slit pores, a realistic model of methane in carbon-based slit pores, and binary mixtures of methane with hypothetical counterparts having different attractions to the solid. The method is seen to be robust and particularly suited for the study of study of transport of dense fluids and liquids in nanoconfined media.

## Introduction

Understanding and modeling of mass transport in porous media is essential in nearly all branches of natural sciences and engineering given the ubiquity of fluid flow in natural and anthropogenic solids. Particularly in engineering applications, a significant number of processes exploit the relative differences in mass transport to separate different components of a fluid mixture. By employing porous matrices, such as membranes, the optimal design of separation units can lead to significant reductions in cost and energy consumption. An early example of separation of fluids using porous media is water desalination using reverse osmosis membranes, where salt is removed from water without the need for energy-intensive distillation units.^1^ Since the turn of the century, advances in technology and a perennially increasing control over nanostructure design has led to the production of porous materials with remarkable adsorption and transport properties, sometimes counterintuitive or unexpected. Fluids flowing within carbon nanotubes (CNTs)^2−5^ exhibit extremely large fluxes. Structured solids such as zeolites^6,7^ and metal–organic frameworks (MOFs),^8−10^ with very large surface area to volume ratios, have the potential of being selective to certain molecules due to a combination of steric and energetic effects, which makes them ideal for separation and catalysis processes. Extremely thin yet strong polymer membranes have been designed to be used in nanofiltration^11,12^ and more recently polymer membranes have been used as an alternative to distillation in the fractionation of crude oil.^13^ The issues associated with the production of oil through unconventional tight nanoporous shale rocks is another example of the unforeseen behavior drawn by the flow of fluids through ultraconfined media.^14^

Optimal design in separation processes has an enormous impact on overcoming current scientific and environmental challenges.^15^ In many instances, given the nanoscale porosities of these materials, understanding the precise mechanism of mass transport is challenging. It is not known to what extent traditional kinetic and phenomenological models can be used to predict transport in these materials. Generally speaking, there are three theories used to characterize transport in porous materials. The first is based on Fick’s law, which employs an empirical constant to relate the transport (mass flux) to the macroscopic density (or pressure) gradient that drives it.^16^ The strategy is conceptually straightforward and commonly implemented in the analysis of experimental results. The resulting coefficients suffer from many drawbacks, namely, a lack of transferability, and a nontrivial pressure, temperature, and concentration dependence. The Onsager formulation, based on irreversible thermodynamics,^17^ recognizes that the driving force for transport is actually a gradient of chemical potential. The transport coefficients thus generated are fundamentally robust and better behaved than the Fickian counterparts, but they are conceptually challenging as chemical potentials cannot be directly measured. A final formulation, defined as the generalized Maxwell–Stefan theory,^18^ suggests the description in terms of so-called corrected diffusities. There is a formal link between all these formulations.^19,20^ However, in all of these descriptions, there is a need to experimentally determine the transport coefficients, i.e., there are no currently available fully predictive methods.

Experimental determination of mass transport and diffusion in nanoporous materials is challenging.^21,22^ A fundamental predictive theory behind transport in nanoconfined spaces is lagging behind the bulk–fluid counterpart, mainly due to the complexities of incorporating the surface–fluid effects, which are, in many cases, dominant.^23^ Thus, as a complementary tool to experiment and theoretical modeling, molecular simulations can provide useful insight into the mechanism of transport and separation in confined media.^23^

Molecular dynamics (MD), Monte Carlo (MC), and a combination of both are commonly used in literature to study transport of pure fluids and multicomponent mixtures.^24−26^ Calculations can be based on the analysis of systems in or away from equilibrium.^27^ In the equilibrium molecular dynamics (EqMD) method, the mean squared displacement, or velocities, of individual particles and their centers of mass are used to measure the motion of the fluid. To calculate transport diffusivities, additional information, namely, adsorption isotherms, are included. Otherwise, the tracking of particle positions or velocities is employed to predict transport diffusivities of multicomponent mixtures.^18,28−32^ A particularly relevant scenario to this manuscript is the calculation of transport diffusivities in nanopores.^33−36^

Calculating transport diffusivities using equilibrium molecular dynamics requires very long simulation runs,^37^ and additional simulations (typically Grand Canonical MC) to calculate the Darken correction factors from adsorption isotherms (∂μ/∂(ln ρ)) are required. As an alternative, nonequilibrium molecular dynamics (NEMD) techniques have been developed measuring transport by inducing a flux by external fields or by artificial gradients.^27,38^ One of the first reported nonequilibrium methods is the external-field NEMD (EF-NEMD) method of Evans and Morris,^39^ where an external field exerts a force on all fluid particles, generating a steady-state nonequilibrium flux. The ratio of the flux to the force is identified as the inherent transport coefficient or Onsager coefficient. This method has been successfully used to study pure systems^27,38^ and binary mixtures.^40−42^ A similar method of using walls to push fluid particles through pores in a pistonlike fashion has also been proposed by Wang et al.^43,44^ To report transport diffusivities using these techniques, adsorption isotherms are required to calculate the Darken factors (see the “Methodology” section).

Other NEMD methods have been developed that do not require adsorption isotherms to measure transport diffusivities. These methods rely on having density gradients across the pore, with the ratio of the induced flux to the density gradient being the transport diffusivity. One of the earliest of such direct measurement methods is the gradient relaxation molecular dynamics (GRMD)^38^ method where a system is initially set up with a density gradient and is then allowed to relax until fully equilibrated. The evolution of the fluid motion to an equilibrated state is then modeled by the diffusion partial different equation to measure the transport diffusivity. Given that the system never reaches steady state until fully equilibrated, it can be difficult to measure the transport diffusivity using ever changing density gradients and fluxes, leading to poor statistics. More efficient algorithms have been introduced by ensuring a steady-state gradient, and thus a steady-state flux, reducing the error in measured variables. One of the most common nonequilibrium steady-state methods is the dual control volume grand canonical molecular dynamics (DCV-GCMD) that has been used to measure transport diffusivities of pure fluids^27,45−48^ and mixtures.^49,50^ DCV-GCMD combines insertion and deletion methods of grand canonical MC with MD so that particles can be deleted downstream of the pore and inserted upstream to create a steady-state chemical potential and density gradient across the pore. The combination of MC with MD has clear advantages, as a steady-state gradient can be achieved and the pressures upstream and downstream can be clearly defined. However, the use of both MC and MD moves can be cumbersome with particle insertions being particularly difficult for high pressures and dense fluids. A more recent method fixing chemical potentials across a pore is that of Ozcan et al.^51^

Other NEMD techniques exist that do not rely on chemical potentials, and subsequently on insertions and deletions. One relevant approach is that of Li et al.,^52^ where a pressure gradient is achieved using a partially reflecting membrane. A membrane is positioned in the simulation box where particles crossing the membrane in a certain direction cross it without hindrance, yet particles attempting to cross the membrane in the opposite direction have a probability of being reflected back. This leads to a pressure gradient and a steady-state flow.

Finally, the boundary-driven NEMD (BD-NEMD) method of Frentrup et al.^37^ is a unique method of measuring transport diffusivities where MD is solely used to induce steady-state density gradients across nanopores. This is done by applying an external field only to a small region in the simulation box, positioned far from the pore. This tool has been extensively used in literature for pure components^37,53−57^ and mixtures.^58,59^

Although the BD-NEMD method has been extensively used in literature, it is not known to what extent transport diffusivities measured in this technique agree with other proven methods, such as the EqMD method. Moreover, there are significant assumptions that need to be addressed to ensure that transport diffusivities measured from the BD-NEMD and EqMD methods agree under all conditions. Moreover, although this method has been used to study multicomponent mixtures,^58^ only the self-transport coefficients have been measured, and the effect of the cross-species transport coefficients have not been studied. The values of the cross-species transport coefficients have not been previously measured using the BD-NEMD method, and it is not known to what extent transport diffusivities of multicomponent mixtures measured using this method agree with those from EqMD simulation. This work aims to address these issues.

## Methodology

Several excellent reviews discuss the relationship between the different transport coefficients which may be directly or indirectly measured in molecular simulations. The reader is referred to them for extension on this topic.^19,60^ For completeness, we will briefly discuss the most relevant expressions and relationships.

### Transport under Equilibrium: Benchmark Case

In molecular simulations, a common practice is to measure the motion of molecules using the self-diffusivity of individually tagged particles of type *l*, which can be calculated using the Einstein relation:^61^
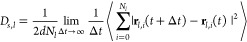
1where **r**_*l*,*i*_ is the vector describing the position of the *i*th particle of molecular type *l*, *N*_*l*_ is the total number of molecules of type *l*, and *d* is the dimension of the vector **r**_*l*,*i*_. In bulk, *d* has a value of 3 as particles can move in all three dimensions. However, inside porous materials, the value of *d* can range from 1 to 3, depending on the dimensions where particles can freely move. The angle brackets refer to an ensemble average.

Although self-diffusivity is informative in describing the motion of individual particles, it does not describe the *collective mobility* of molecules, i.e., how the collective (center of mass) motion of molecules of type *l* is related to the collective motion of all molecules of type *m*. Given that flow in porous materials is a consequence of the collective motion of molecules, self-diffusivity cannot be used to calculate transport of fluids apart from extremely dilute systems where particles do not experience strong intermolecular forces and thus move independently.^62^ A more general equation is thus used to describe the transport of particles of type *l*, influenced by the collective motion of particles of type *m*, denoted by Λ_*lm*_:^35,38,63^
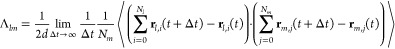
2

For a pure component system, Λ_11_ corresponds to the Maxwell–Stefan (MS) diffusivity, *D̵*_MS_, also known as the collective, or Darken corrected, diffusivity, *D*^c^. Thus, eq 2 can be simplified for a pure component system:
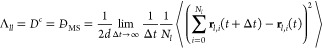
3

For multicomponent mixtures, elements of the matrix [**Λ**] are related, but they are not identical to the exchange coefficients of the MS diffusivity. Λ_*ij*_ provides an indirect route to calculating transport diffusivities of multicomponent mixtures (eq 11). In addition to the Einstein relation, Λ_*ij*_ can also be measured using Green–Kubo relations which use velocities instead of displacements.^29^ Although very commonly used, calculating collective diffusivities from equilibrium molecular dynamics simulations requires many independent simulations (or one very long simulation) to get acceptable statistics.^33,62,64^ This renders this method laborious and computationally inefficient.

Moreover, in order to compute transport diffusivities from equilibrium simulations, additional information is required in the form of the adsorption isotherms for the fluid in the porous material. While the equilibrium methods are accepted as the *de facto* “gold standard”,^65^ they are computationally inefficient. It is in this space that nonequilibrium models can be an alternative to calculate transport diffusivities.

In the next section, we will discuss an implementation of a boundary-driven nonequilibrium method to binary mixtures and the relationship between the transport coefficients obtained as compared to those derived from other routes.

### Transport under Non-Equilibrium

Transport diffusivity, *D*^*t*^, is the mass transport coefficient used in the continuity and Fick’s equations, relating the flux of a given species, *i*, in a porous medium to its concentration gradient:^36,40,66^
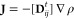
4Here, **J** is the *n* × 1 vector of fluxes of *n* components inside the pores, [**D**_*t*_] is the *n* × *n* Fick or transport diffusion matrix, and ∇**ρ** is the *n* × 1 vector of density gradients. It is important to note that fluxes need to be measured relative to a specified frame of reference which is the porous solid and is assumed to be stationary. Moreover, it is also important to mention that ∇**ρ** corresponds to the density gradient of the fluid inside the porous media, i.e., the density of the adsorbate.

For a binary mixture where the flux is measured in one direction, *z*, the above equation can be written as^67^
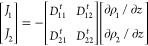
5where *D*_*ii*_^*t*^ is the self-transport diffusivity, i.e., the contribution to the flux of species *i* due to its own concentration gradient, and *D*_*ij*_^*t*^ is the mutual diffusivity, corresponding to the contribution to the flux of species *i* due to the concentration gradient of species *j*. For a binary mixture, none of the elements of the transport diffusivity matrix are necessarily similar to each other, i.e., *D*_*ij*_^*t*^ ≠ *D*_*ji*_^*t*^.

Crucially, a concentration gradient is not the only source of mass flux, e.g., the Soret effect^68^ describes the flux of mass driven by a thermal gradient which has been studied in bulk^68,69^ and under confinement^70−72^ using simulations. Similarly, consider a system in vapor–liquid equilibrium where there is a clear gradient in density; however, there is no net mass flux across the interface. As an alternative to the incongruities of the Fickian formulation, Onsager’s treatment provides a fundamental starting point relating the fluxes to the underlying mass transport driving force. In an isothermal case, one could relate the fluxes to the gradients of the chemical potential difference of each species, ∇**μ**:^17^
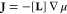
6Here, [**L**] is the symmetric matrix of Onsager coefficients (phenomenological coefficients). An interesting inference of the Onsager treatment is that the flux of species *i* becomes dependent on the chemical potential gradient of both components *i* and *j*. There is an explicit recognition within this formulation that there are cross-component effects, i.e., that the flux of one component may have an impact on the flow characteristics of the other. In particular, the cross coefficient terms become important because the density of the system becomes liquidlike and correlation effects are strong.^73^ For a binary mixture
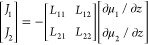
7where [**L**] is symmetric
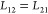
8which is not true for mutual transport diffusivities. The matrix [**Λ**] with elements Λ_*ij*_ (eq 2) is directly related to the Onsager matrix [**L**]. One can redefine the transport coefficients for a binary fluid system flowing through a porous media as follows:^73^
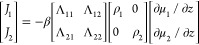
9where β is 1/*RT* where *R* is the gas constant and *T* is the temperature. Commensurate with eq 5, ρ_*i*_ is the density of the adsorbed fluid. The Onsager coefficients, *L*_*ij*_, do not have the units of *length*^2^/*time* customary to describing transport, while the modified term Λ_*ij*_ has the same units as both transport and self-diffusivities. Going a step further, and using the Jacobian matrix for a chain of variables, eq 9 can be modified so that the driving force is given in terms of the adsorbed density gradients:
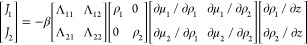
10

Comparing eq 5 with eqs 7 and 10, the relationship between the diffusion coefficients may be expressed as
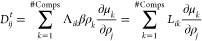
11and
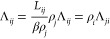
12where #Comps is the number of components. The term βρ_*i*_∂μ_*i*_/∂ρ_*j*_ is commonly known as the Darken factor, Γ_*ij*_, and for a pure system, it is the proportionality constant relating transport to the collective diffusivity:
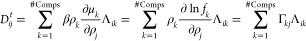
13The Darken factor can be calculated from adsorption isotherms obtained from simulations or experimental data.

A critical review of some of the alternative techniques for calculating diffusion coefficients can be found in literature.^17,27^ Of particular note is the Maxwell–Stefan formalism.^18,74−76^ These models are all “equivalent” and the relationship between them has been presented elsewhere.^16,77^

### Boundary Driven Non-Equilibrium Molecular Dynamics

**Figure 1:**
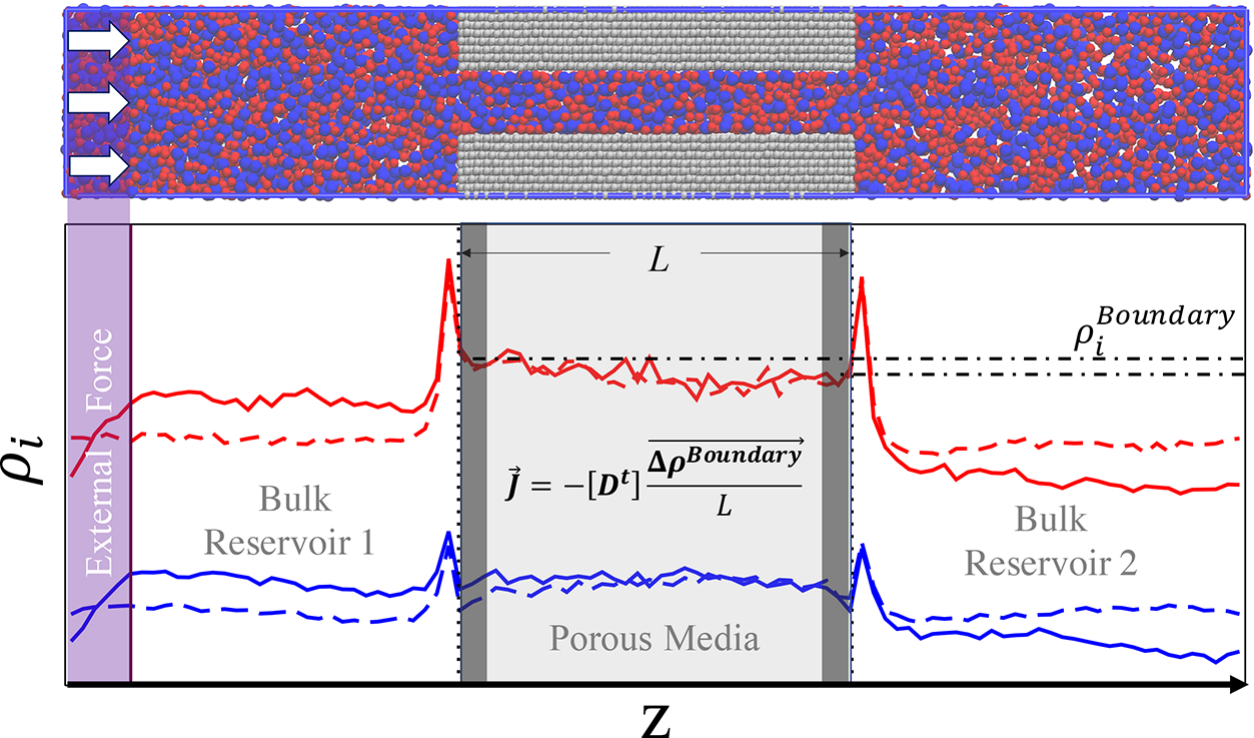
BD-NEMD method employed, as applied to a binary mixture. Top: Snapshot from the simulation, indicating the direction of flow, with different fluid species colored red or blue and the fixed pore colored gray. Arrows denote the regions where an external acceleration is imposed. Periodic boundary conditions is employed between left and right reservoirs. Bottom: Concentration profile of each species is color matched with the simulation snapshot. Dashed lines correspond to systems with no external forces, and solid lines are the steady-state result after applying external forces resulting in concentration gradients across the pore. Pore boundary regions are assumed to be in local equilibrium with neighboring bulk regions.

**Figure 2:**
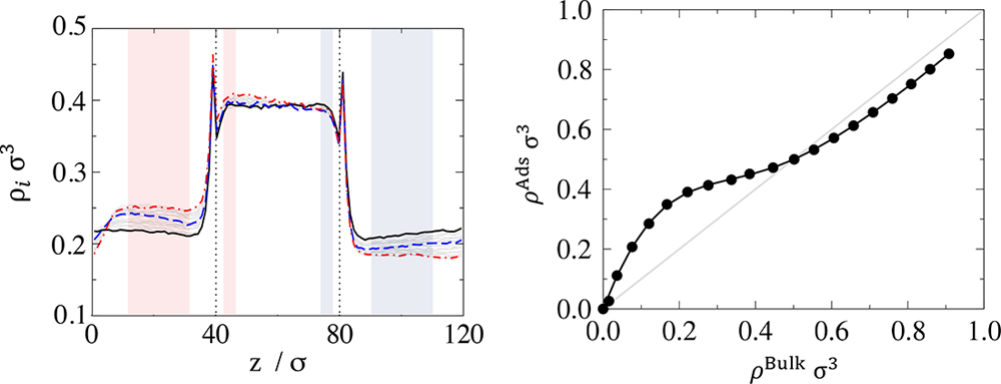
LJ fluid at *T* = 1.5 ε/*k*_B_, flowing through a FCC pore with pore height *H* = 20/3 σ, with the solid particles being the same as the fluid LJ particles. (left) ρ vs z using 20 different forces. All cases are plotted gray except for three: no force (black solid), highest force (red dashed and dotted), and medium force (blue dashed). To calculate density gradients inside the pores, the local equilibrium assumption is invoked, and the adsorbed densities at the boundaries (highlighted) are estimated using bulk densities from equilibrium simulations. (right) Adsorbed vs bulk densities of the same system used to estimate the adsorbed densities at the boundaries of the pore. Errors are within the size of the symbols.

Given a density gradient inside a pore and an appreciable flux, it is possible to directly assess the transport diffusivity of a fluid flowing inside a pore. To induce such gradient, we revisit the proposal of Frentrup et al.^37^ as applied to pure fluids, modifying key assumptions, and extending it to multicomponent mixtures. First, a simulation box composed of two bulk reservoirs in contact with the pore is set up. Fluid particles are added until a target global density is reached and the system is left to equilibrate. An external field is then applied in a small section of the simulation box, which in this work is 2 nm wide, far enough from the pore. This region is also the boundary of the two reservoirs. This external field exerts a directional force on all particles within the small region, pushing particles at the boundary of the two reservoirs to one side, thus increasing the concentration of the fluid in one reservoir, and depleting the amount of fluid in the other. The reason the external force is applied far away from the pore is to minimize the effects of the applied force on the flow across the pore, to ensure that the flow is being induced by the density gradient across the pore. The system eventually reaches steady state, and a steady-state concentration gradient occurs within the pore. This is illustrated in Figure 1 for a binary mixture. In terms of implementation, the directional force is the result of applying an acceleration to the particles. In this work, the same acceleration is applied to all species. Interestingly, in this method there are two bulk regions on each side of the pore. For small applied forces, the density of each bulk region remains fairly constant, and the observed density gradient is only within the pore. Applying different external forces to the boundary leads to different bulk compositions and different density gradients across pores, as can be seen in Figure 2, for a pure fluid flowing through a slit pore.

In Figure 2 (left), different forces are applied to the fluid and a density gradient develops within the pore. It can be clearly observed that the density in the adsorbed phase and in the bulk are very different. This implies that density gradients across the pore, and consequently the transport diffusivities, are different dependent on which density (bulk or adsorbed) is used. It is important to mention that commonly in literature using BD-NEMD transport coefficients are calculated using reservoir densities^37,54,56,78,79^ which is only rigorous if the adsorbed densities are the same as bulk densities, i.e., for nonadsorbing systems. As will be shown later, an incorrect choice leads to significant discrepancy between measurements of transport diffusivities using the EqMD and NEMD methods.

Therefore, a key modification of the BD-NEMD method implemented in this work is to use density gradients inside the pore instead of calculating the density gradients using bulk (reservoir) concentrations. This can be particularly challenging, as the statistics inside the pore can be poor. In order to estimate the density gradients more robustly, an assumption is made that inside the pore (pore entrance and exit), the boundaries are at local equilibrium with the adjacent bulk reservoirs; thus, the amount adsorbed at the boundaries can be calculated from equilibrium simulations of a bulk region. Knowing the bulk densities on each side, the adsorbed density at the boundary inside the pore can be estimated, and the density gradient is calculated. An example of the such relationship between bulk and adsorbed density can be seen in Figure 2 (right).

Once the density gradient is calculated, the flux of each component, *J*_*i*_, is calculated in the middle of the pore:
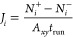
14where *A*_*xy*_ is the area of the plane in the pore perpendicular to the direction of the flow, *t*_run_ is the total simulation running time, and *N*_*i*_^+^ and *N*_*i*_^–^ are the total number of particles of species *i* that have passed the middle of the pore in the same and opposite direction to the direction of the external force, respectively. The choice of the middle of the pore is arbitrary if the cross-sectional area of the pore does not change.

By running simulations with different forces, one could obtain different density gradients and fluxes which can be used to improve the statistics. Transport diffusivities can be assessed using the following equation:
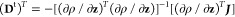
15

For an *m* component mixture, **D**^**t**^ is the transport diffusivity matrix defined in eq 5, and **∂ρ/∂z** and ***J*** are *n* × *m* matrices of concentration gradients and fluxes, respectively. *n* is the number of *in silico* experiments carried out, each with a different boundary force, and the superscript “*T*” denotes a matrix transpose.

One of the benefits of using the BD-NEMD technique over other nonequilibrium approaches is the fact that the force itself is not used to evaluate transport coefficients. It is applied in a region so far away from the pore that it does not affect the transport inside the pores. However, what the force does is to help in building up fluid on one side of the pore. Transport is a thus a consequence of the concentration gradient across the pore, and not the applied force.

Thus, it does not strictly matter what force is applied to either species, as long as there are measurable concentration gradients and fluxes of all species across the pore. If fluxes are only functions of concentration gradients, and not applied forces, then transport coefficients are independent of those concentration gradients and thus different forces can be applied to any species.

This is in contrast with other NEMD techniques, such as the external force NEMD (EF-NEMD) method, where a force is applied to all species. In that case, the applied force is directly responsible for the transport of both species and transport coefficients are calculated by relating fluxes to applied forces. In that case, if molecules have different masses, then one might observe buoyancy effects and artifacts that might affect the measurement of transport coefficients. To overcome that particles having different masses, accelerations should be modified to ensure the same force applied to different species.

### Molecular Interactions

All fluid molecules are modeled as single spheres, with the Mie potential describing intermolecular interactions between particles. Cross interactions are resolved by using the Lafitte et al. combination rules.^80^ Details are provided in the Supporting Information.

### Modeling Adsorption

As highlighted previously in Figure 2 (right), in the BD-NEMD method it is important to relate bulk densities to the adsorbed densities. Moreover, if one wants to calculate transport diffusivities from the EqMD method (eqs 2 and 13), then adsorption isotherms relating adsorbed densities to chemical potentials or fugacities are essential.

To relate adsorbed densities to bulk densities, in this work both grand canonical Monte Carlo (GCMC)^61,81^ and EqMD simulations have been used. The GCMC method models the pore and the bulk separately using a defined chemical potential, whereas the EqMD method simultaneously models a bulk in equilibrium with the pore. For each GCMC simulation, 40 000 cycles were used, where each cycle consists of a displacement move, an insertion, and a deletion. For particle displacement moves, the probability of success was set to 0.25.

At higher densities, the GCMC method can become less efficient, as insertion and deletion of particles is more difficult. A particular advantage of the BD-NEMD method proposed is that the same system set up can be used to measure adsorbed densities by turning the external forces to zero, removing additional burden of setting up new simulations.

To calculate chemical potentials, for this particular force field one may use directly a molecular based equation of state (EoS), SAFT-γ Mie.^82^ The inputs of this EoS are the same as the Mie potential:
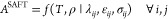
16where *A*^SAFT^ is the molar Helmholtz free energy of the fluid. The correspondence between the results of the equation of state and those from molecular simulations has been discussed elsewhere.^83−90^

An additional advantage of employing the SAFT-γ Mie EoS alongside the BD-NEMD method is that it allows accessing Λ_*ij*_ (eq 2), given the condition that the boundaries of the pore are in equilibrium with the bulk regions:
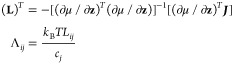
17where ∂**μ**/∂**z** is either calculated using SAFT-γ Mie EoS or from GCMC.

#### Adsorption of Multicomponent Mixtures

Although GCMC and EqMD could be used to calculate adsorbed densities of binary mixtures, given the additional degree of freedom stemming from considering the compositions, the pure component adsorption isotherms of each component are used as input to predict the adsorption of multicomponent mixtures through the ideal adsorption solution theory (IAST).^91,92^ In this paper, the IAST method is implemented using the pyIAST package, where the pure component adsorption isotherms (pressure vs adsorbed concentration) are used as inputs.^93^ Details are provided in the Supporting Information.

### Systems Studied

Four case studies are chosen in this study: (i) Case I: A pure LJ fluid within a slit pore; as a validation of the proposed BD-NEMD method by comparison to transport diffusivities calculated using EqMD. (ii) Case II: Pure methane within a slit pore; as an example of application to realistic fluids. (iii) Case III: A binary mixture of two methane-like fluids, with one having realistic parameters (as in Case II) and the other having augmented interactions with the pore; as an example application on the effect of the solid–fluid interaction on selectivity. (iv) Case IV: A binary LJ mixture, where one species is the same as Case I, and the other has a radius 30% larger; as an example application on the effects of size differences on self-and mutual diffusivities in binary mixtures

All parameters for the four systems studied are presented in Table 1. All walls are modeled as FCC lattices, and for all cases, the lattice constant is .

**Table 1:** Force-Field Parameters for the Four Cases Studied

Case I				
*T* = 298 K		*H* = 2.0 nm		
species		interaction parameters		
*i*	*j*	λ_*ij*_	ε_*ij*_/*k*_B_/K	σ_*ij*_/nm
LJ fluid (LJF)	LJF	12.0	200.0	0.300
LJ wall (LJW)	LJW	12.0	200.0	0.300
LJF	LJW	12.0	200.0	0.300
*M*_w,LJ_ = 40.00 g mol^–1^				

Case II				
*T* = 298 K		*H* = 2.6 nm		
species		interaction parameters		
*i*	*j*	λ_*ij*_	ε_*ij*_/*k*_B_/K	σ_*ij*_/nm
methane (M)	M	16.4	170.8	0.375
wall (W)	W	12.0	170.8	0.350
M	W	14.0	170.4	0.363
*M*_w,M_ = 16.04 g mol^–1^				

Case III				
*T* = 298 K		*H* = 2.6 nm		
species		interaction parameters		
*i*	*j*	λ_*ij*_	ε_*ij*_/*k*_B_/K	σ_*ij*_/nm
methane A (MA)	MA	16.4	170.8	0.375
methane B (MB)	MB	16.4	170.8	0.375
wall (W)	W	12.0	170.8	0.350
MA	MB	16.4	170.8	0.375
MA	W	14.0	170.4	0.363
MB	W	14.0	136.4	0.363
*M*_w,MA_ = *M*_w,MB_ = 16.04 g mol^–1^				

Case IV				
*T* = 298 K		*H* = 2.0 nm		
species		interaction parameters		
*i*	*j*	λ_*ij*_	ε_*ij*_/*k*_B_/K	σ_*ij*_/nm
LJF-1	LJF-1	12.0	200.0	0.300
LJF-2	LJF-2	12.0	200.0	0.400
LJ wall (LJW)	LJW	12.0	200.0	0.300
LJF-1	LJF-2	12.0	193.9	0.350
LJF-1	LJW	12.0	200.0	0.300
LJF-2	LJW	12.0	193.9	0.350
*M*_w,LJF-1_ = 40.00 g mol^–1^, *M*_w,LJF-2_ = 94.81 g mol^–1^				

### Simulation Details

In all simulations, a system is set up with a solid pore of *L*_*z*_ = 12 nm positioned in the middle of a 36 nm simulation box, thus having two bulk regions on each side, each being 12 nm in length. The pore region is an FCC smooth slit pore. Details of its structure and the definition of the pore height are given in the Supporting Information. Lengths of the box (and the pore) in the *x* and *y* dimensions are the same, being greater than 10 σ. The large values of pore dimensions and simulation box sizes are chosen to minimize finite size effects. In particular, the ratio of particle size to pore length (σ/*L*) is always less than 0.05, and the ratio of pore height to pore length (*H*/*L*) is always less than 1. From previous studies,^94^ it is known that these dimensions are far from those resulting in significant finite size effects.

The total void volume of the simulation box is known and fixed, and particles are added to a target global fluid density. Equilibrium molecular dynamics simulation are then run in the *NVT* ensemble. Thus, the system equilibrates, and the adsorption isotherms can be assessed.

With the system equilibrated, the final configuration is used for two purposes. First, the same system is used as the initial configuration for the BD-NEMD simulations. Second, the pore section of the final configuration is isolated (the bulk regions removed and periodic boundary conditions imposed in the *x*- and *z*-directions) and used in EqMD simulations to calculate Λ_*ij*_ in an essentially infinite pore setup. The EqMD and NEMD simulations are then used to calculate transport diffusivities which were compared.

Each simulation is run for 10 million time steps of 2 fs. The first 4 ns are used for equilibration, or reaching a steady state, and the remaining 16 ns were analyzed as the main production run.

For both the BD-NEMD and EqMD methods, following previous studies,^37,57^ simulations were run in the *NVT*_W_ ensemble, where *T*_W_ refers to the temperature coupling of the solid particles only. This means that no temperature coupling is used for the fluid as to avoid influencing its dynamics, and instead energy was added or removed using wall particles as a thermostat, i.e., the excess energy input of the external force is removed by the walls. To implement this, the pore solid particles are allowed to vibrate about their equilibrium position using position restraints of the harmonic form, with a bonding potential of 10 000 kJ mol^–1^ nm^–2^ and an equilibrium bond distance of σ_wall_. By only applying temperature coupling to the solid particles, the temperature of both solid and fluid are kept constant about the equilibrium temperature, which can be found in the Supporting Information. Moreover, to ensure the positions of the fluid were not modified, no center of mass motion removal was used.

The BD-NEMD simulations are run using a modified version of GROMACS/5.1.2^95^ and for each simulation at each state point, 20 different simulations were run using applied accelerations in the range of 0–0.004 nm ps^–2^ to keep the perturbations of the system within the ”linear regime”. EqMD simulations are run using GROMACS/2018^96^ to ensure results are not subject to software bias. All visualizations of simulations have been rendered using the VMD package.^97^

## Results and Discussion

### Case I: Pure LJ Fluid

For the pure LJ system in a slit pore of height 2 nm (6.67 σ), simulations are run from the dilute limit to highly concentrated systems, i.e., ρσ^3^ = 0.02–0.85 at *T* = 1.5 ε/*k*_B_, which is above the critical temperature of the LJ fluid (*T*_c_ = 1.31 ε/*k*_B_).^98^ The coexisting fluid density at the supercritical fluid–solid transition for a bulk LJ fluid is at ρσ^3^ = 1.015^99^ which set the upper limit of densities to 0.85, ensuring the fluid inside the pore did not undergo a phase transition to a solid phase.

Figure 2 (right) shows the measured adsorbed concentrations for all bulk concentrations studied. It could be clearly seen that the pore is very selective at lower densities, while at higher densities the concentration is only around five percent less than bulk concentrations. The latter underestimation is presumably an artifact of the particular definition of the pore height used in this work.

#### Adsorption Isotherms

The BD-NEMD method can be used to measure both transport diffusivities (eq 15) and collective diffusivities, Λ_*ij*_, (eq 17). While the first calculation is direct, for the latter, one needs to find the chemical potential gradients across the pore. Otherwise, Λ_*ij*_ can also be directly calculated using EqMD (eq 2). As a test of robustness of the BD-NEMD method developed, Λ_*ij*_ is compared with both methods.

**Figure 3:**
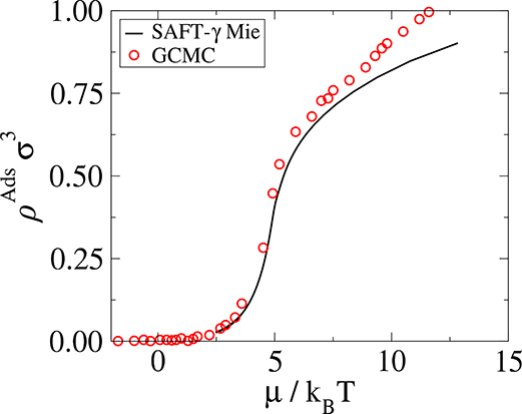
Adsorption isotherm of Case I. The SAFT-γ Mie approach uses direct MD simulations where the pore and the adsorbed fluid are in equilibrium with the bulk, and chemical potentials are estimated using the equation of state with the bulk densities as input. Errors are within the size of the symbols.

Furthermore, chemical potentials are required to evaluate the Darken factors, so Λ_*ij*_ could be used to calculate *D*^*t*^ (eq 13). Chemical potentials are assessed using both GCMC and the SAFT-γ Mie EoS. While using the EoS, the chemical potential is calculated using the bulk density at equilibrium with the adsorbed density, whereas in GCMC (μ*VT* ensemble) chemical potential is an input from which adsorbed densities can be determined. Although the two methods are fundamentally different to each other, a quantitative agreement between them can be seen in Figure 3. The disagreement at higher densities is attributed to sampling inefficiencies in GCMC. The equation of state is computationally much more efficient; thus, the SAFT-γ Mie EoS is chosen as the preferred method of measuring chemical potentials and Darken factors.

The relationship between chemical potential and adsorbed densities can be used to calculate chemical potential gradients across the pore from the BD-NEMD simulations, which can then be used to determine Λ_*ij*_ using eq 17. Additionally, the Darken correction factor is also calculated from the same relationship:
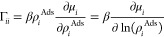
18

**Figure 4:**
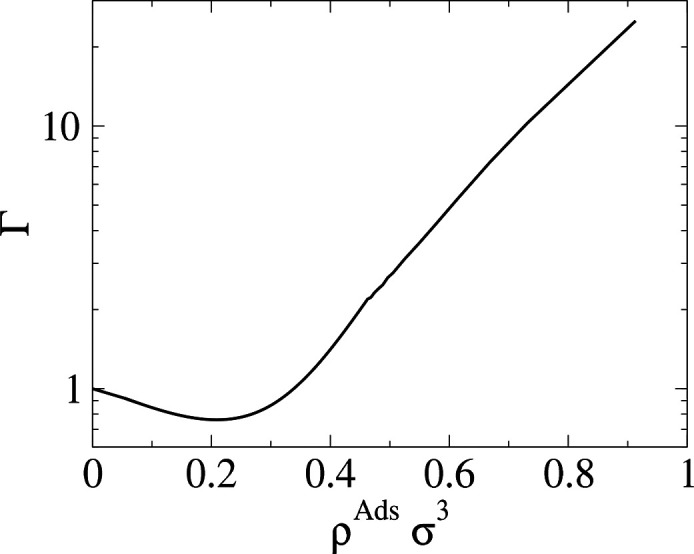
Darken factor, Γ, vs adsorbed density, ρ^ads^ calculated from the SAFT-γ Mie EoS.

Γ_*ii*_ is calculated as a function of the adsorbed concentration using SAFT-γ Mie EoS, and the results can be seen in Figure 4 (right). The Darken factor approaches the value of 1 at infinite dilution, corresponding to the expected value for an ideal gas. As more particles are added at lower densities, the presence of attractive forces lead to a decrease in the value of the Darken factor. The Darken factor increases as further insertion of fluid particles becomes less favorable, culminating in very large increases in the chemical potential. The density profile inside the pore can be seen in the Supporting Information. At low densities, there is only a small layer of adsorption nearest to the surface. At high density, the pore is saturated, and the fluid is highly ordered. The onset of saturation occurs at ρσ^3^ = 0.4, after which particle insertions become less favorable and the chemical potential increases.

#### EqMD

**Figure 5:**
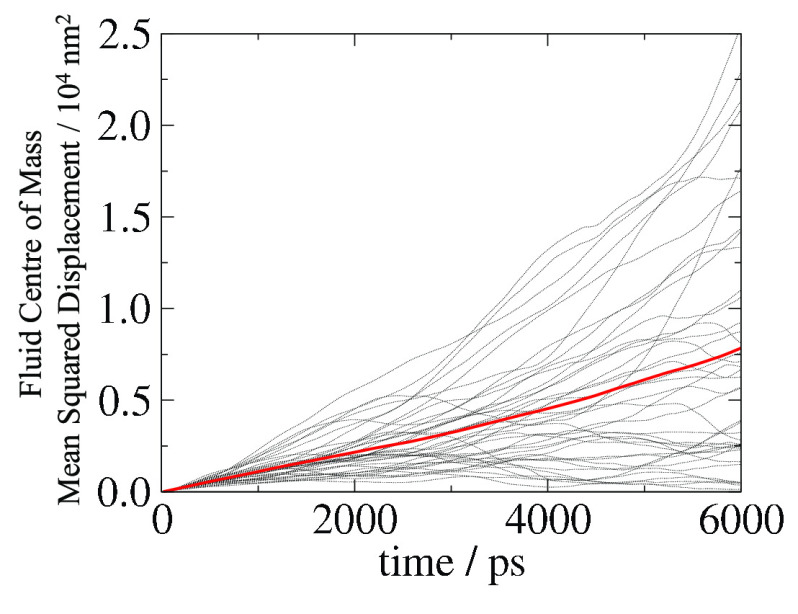
Centre of mass mean squared displacement of 60 different EqMD simulations of the same system (ρσ^3^ = 0.5). Each dotted line represents results of one simulation. The average of the 60 simulations is highlighted as the thick red line which is related to the collective diffusivity, Λ.

For each value of the density of fluid inside the slit pore, 60 independent equilibrium simulations, or 300 million time steps in total, were run. Self-diffusivities were calculated using eq 1. Then, the mean square displacement (MSD) of the center of mass of the fluid in the two dimensions (plane) parallel to the pore surface is measured as a function of time. As can be seen in eq 2, in the limit of an infinite time, collective diffusivity, Λ_*ii*_ is given by the slope of the linear line describing MSD as a function of time. Figure 5 shows the results for one density (ρσ^3^ = 0.5), and although the average value of all 60 simulations is linear, each simulation has a drastically different MSD profile relative to the average. Thus, the errors associated with measuring collective diffusivities can be particularly large. This is found to be true across all concentrations.

With the collective diffusivities measured from EqMD and the Darken factors from adsorption isotherms, it is possible to calculate transport diffusivities, *D*^*t*^ = ΓΛ (eq 11).

#### NEMD

**Figure 6:**
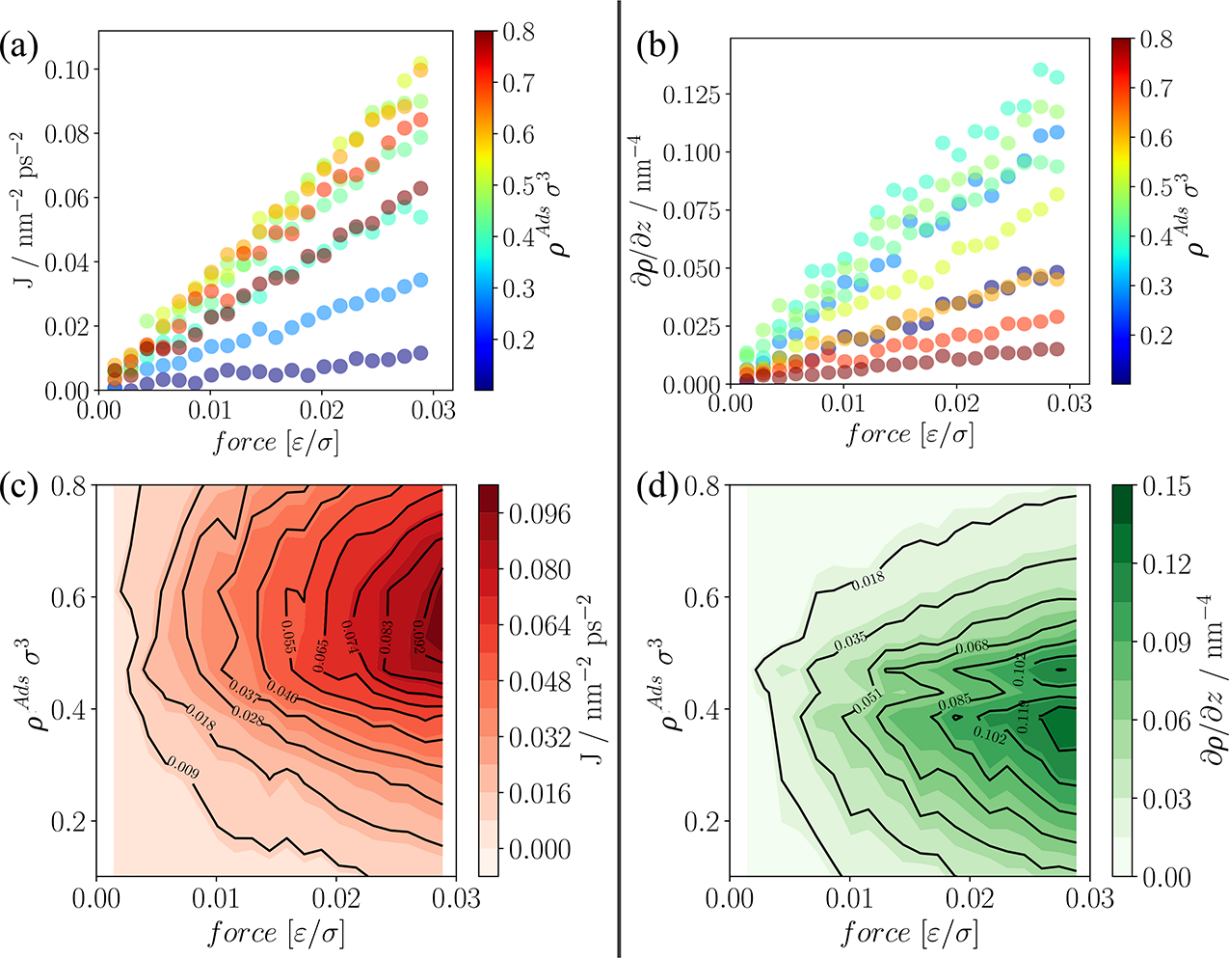
Flux, *J*, and density gradient, ∂ρ/∂*z*, for fluid densities and external forces applied for the LJ fluid. (a) *J* vs force and (b) ∂ρ/∂*z* vs force, where different colors correspond to different fluid densities at equilibrium. (c and d) Contour maps of fluxes and density gradients respectively, showing maxima at intermediate loadings. Errors are within the size of the symbols.

For different densities, 20 different forces are applied at the boundary and fluxes and concentration gradients are evaluated. As can be seen in Figure 6a,b, there is a linear relationship between the applied force and the flux as well as the density gradient, i.e., the system is kept within the linear response regime. Frentrup et al.^37^ observed a nonlinear response when employing forces an order of magnitude higher than that applied in this work.

**Figure 7:**
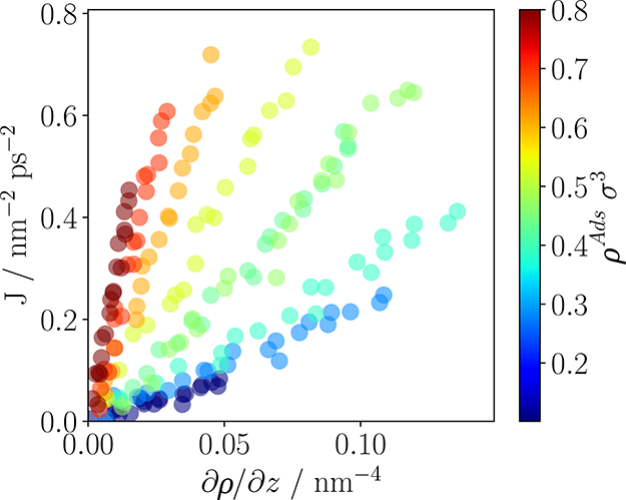
Flux, *J*, vs density gradient, ∂ρ/∂*z* for the LJ fluid. Colors indicate adsorbed density at equilibrium. The slope of each line corresponds to the transport diffusivity for the given loading (eq 15). Errors are within the size of the symbols.

In Figure 6a,b, the fluxes and density gradients are shown for different applied forces, respectively. The symbols are colored based on the equilibrium adsorbed densities, i.e., where the force is null. Figure 6c,d shows the contour maps of fluxes and density gradients as functions of the force and the fluid density. Although one would expect that increasing the external force leads to greater flux and density gradients, for a given applied force the value of flux (or density gradient) peaks at intermediate loadings. This could be explained by noting that at higher densities applying a force to particles at the boundary pushes them into a dense fluid region and hinders the flow. However, this does not mean that the transport diffusivity is highest at intermediate concentrations. The value of flux should not be taken as an indication of faster transport. It is only by relating fluxes to density gradients where a true measure of transport is given, which can be seen in Figure 7. The aforementioned figure clearly highlights that increasing pore loading leads to higher transport diffusivities.

#### Comparison between EqMD and BD-NEMD

**Figure 8:**
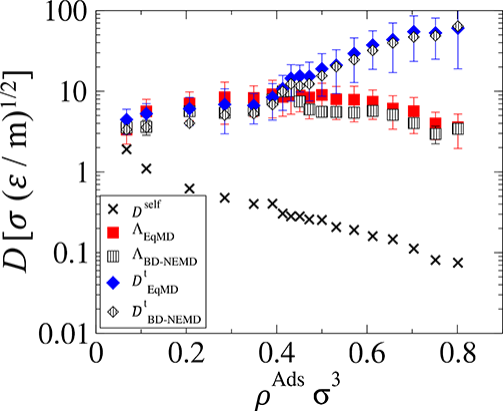
Summary of the different transport coefficients measured for the LJ system studied.

A summary of all transport coefficients measured using the EqMD and BD-NEMD methods is presented in Figure 8. From the EqMD method, self-diffusivity (*D*^self^) and collective diffusivity (Λ_EqMD_) are calculated using eqs 1 and 2, respectively, and transport diffusivity (*D*_EqMD_^*t*^) is calculated using eq 13, i.e., by multiplying Λ_EqMD_ by the Darken factor, Γ. From the BD-NEMD method, Λ_BD-NEMD_ and *D*_BD-NEMD_^*t*^ were calculated using eqs 17 and 15, respectively.

As can be seen, there is quantitative agreement between the BD-NEMD method and the benchmark equilibrium simulations across all densities. The BD-NEMD method captures the same trend as the EqMD simulations; however, both transport and collective diffusivities are underestimated in the nonequilibrium simulation. This underestimation is about 20% in the value of Λ and 15% in the value of *D*^*t*^. The error is greatest at lower densities, which is presumably caused by uncertainties in the equilibrium simulations, as the error bars are very large and the values obtained using the NEMD method all lie within the error associated with EqMD.

It is important to note that while the coefficients approach the same value at infinite dilution, *D*_ρ→0_^*s*^ = Λ_ρ→0_ = *D*_ρ→0_^*t*^ ≈ 3.5 σ(ε/*m*)^2^, upon increasing densities, these coefficients show different trends. Self-diffusivity decreases with increasing densities and is roughly 20 times less at the highest density than at the infinite dilution limit, as particles in denser phases have smaller velocities and less free paths to diffuse uninterruptedly. This trend is not observed in the collective diffusivities, as the value of Λ peaks at intermediate densities of ρ^Ads^σ^3^ = 0.45 and then slightly decreases at higher density (see Figure 6). Given that the Darken factor substantially increases at higher densities, transport diffusivities show the opposite trend to the self-diffusivities being up to 15 times larger at the highest density relative to the lowest. The fact that transport diffusivities can be orders of magnitude larger than self-diffusivities stems from the very significant collective motion of the fluids as a consequence of smoothness of the surface of the slit pore, and any individual movement of adsorbed molecules correlates with the movement of all other molecules in the system.

**Figure 9:**
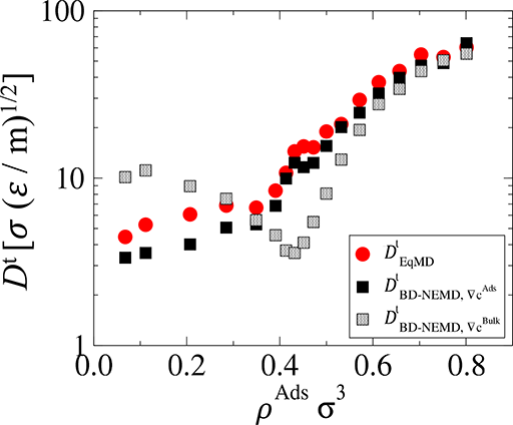
Comparison between transport diffusivities with the BD-NEMD method assuming the concentration gradient is given by the bulk compositions across the pore (open squares), as commonly used in literature, or if adsorbed densities are used (black squares), implemented in this work. Red squares correspond to benchmark EqMD results. Errors are within the size of the symbols.

The key difference between this work and previous BD-NEMD approaches in literature is in the way density gradients are defined in the calculations of the transport diffusivities. As mentioned previously, a common practice is to use the unambiguous bulk reservoir densities. However, as can be seen in Figure 9, using bulk densities as the driving force for calculating transport coefficients inside the porous regions leads to drastically different profile for transport diffusivity as a function of density. Bulk density gradients do not take into account the effect of pore adsorption on transport and/or any pore entrance effects that may be present. Using bulk density gradients leads to significant overestimation of transport diffusivity at low loadings and underestimations at intermediate loadings, with a clear minimum at around ρ^Ads^σ^3^ = 0.4. This minimum is neither observed for the BD-NEMD method developed in this work nor the EqMD benchmark (see Figure 9).

### Case II: Methane

To exemplify how this methodology could be employed to investigate transport of real fluids, the BD-NEMD method is further tested on a system consisting of supercritical methane in a slit pore at *T* = 300 K. Essentially, the system resembles that of Case I but the fluid force field can be traced back to a realistic model. The Mie parameters describing the intermolecular interactions have been previously optimized to reproduce vapor–liquid equilibrium^100^ (see the Supporting Information). The quantitative agreement between results obtained from MD with those obtained from EoS is a unique trait of the SAFT implementation used describing the macroscopic properties of the Mie intermolecular potential, allowing for fast and accurate description of the free energy, and thus chemical potentials of the system.

The top-down coarse-graining technique used to parametrize the force field is effective at producing robust models with transferability and representability, and can be used with confidence to describe transport and adsorption properties.^101−104^ The methane model presented in this work correctly predicts self-diffusivities of supercritical methane at 303 and 333 K at a range of different pressures (see the Supporting Information). The accuracy of the EoS in measuring the self-diffusivities of methane justifies the use of the model in measuring transport coefficients of methane in nanopores.

The slit pore is composed of particles explicitly modeled in an FCC lattice, with self-interaction parameters described using an ad hoc LJ potential, resembling an organic substrate. The height of the pore is 2.6 nm.

**Figure 10:**
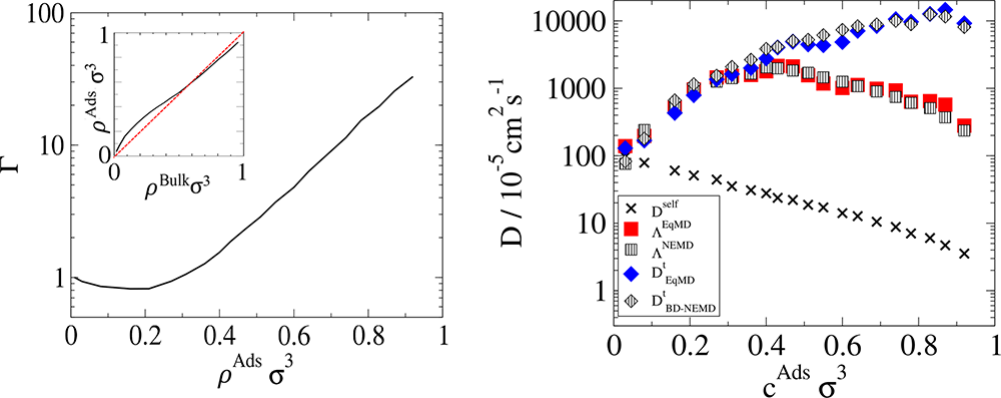
(left) ρ^Bulk^ vs ρ^Ads^ and the Darken factors for methane in 2.6 nm wide slit pore specified in Case II. (right) Summary of diffusivities of pure methane in the pore in Case II. Squares are the collective diffusivities, and diamonds are transport diffusivities. The striped white symbols are from BD-NEMD simulations and blue and red are measured using EqMD.

The relationship between adsorbed and bulk densities and Darken factors is seen in Figure 10 (left) and in the Supporting Information, where it is seen that at low bulk densities the adsorbed densities are higher than the bulk densities. The pore is slightly wider making surface adsorption less pronounced than the adsorption for Case I. Furthermore, relative to the solid particles used in Case I, the solid particles here are larger with a smaller ε, thus leading to weaker attractive energy density, and thus adsorption capacity.

The comparison between the BD-NEMD and EqMD techniques is presented in Figure 10 (right), showing the remarkable agreement between the two methods, with agreements from the dilute limit to very dense fluids spanning orders of magnitude.

### Case III: Transport of a Binary Mixture of Two Methane-Like Fluids with Different Adsorption

Case II was extended by introducing an additional fluid component with similar characteristics to the methane model, henceforth described as MA. This new fluid, MB, has the same intermolecular interactions except that its interaction with the solid pore is less favorable. The cross species energy, ε_*ij*_, was 20% less than that of the MA–solid interaction (Table 1). In the bulk, there is no difference between the two fluids, and the mixture can be seen as having the same bulk properties as pure MA at the same total density. However, the reduction in the value of the cross-species interaction results in slightly different adsorption isotherms, and different pore selectivities.

In order to observe the adsorption behavior of the binary mixture, pure component adsorption isotherms are evaluated and can be seen in the Supporting Information. Ostensibly, the adsorption isotherms of the pure fluids look very similar, but given the different interactions with the pore, in a binary mixture it is expected that the pore would be more selective toward species MA. IAST is used to correlate the adsorption isotherms of the mixture using the pure component adsorption isotherm, estimating pore loadings using bulk pressures and compositions.^91,92^ The predictions of the IAST were then validated against EqMD simulations of binary mixtures, results that can be found in the Supporting Information. Generally, higher partial pressure of each species leads to higher adsorption of said species.

Pore selectivity toward species MA is defined as
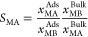
19where *x*_*i*_ is the mole fraction of species *i* and the superscripts “Ads” and “Bulk” refer to compositions inside the pore and in bulk. A value greater than one refers to a pore that is more selective toward species MA.

**Figure 11:**
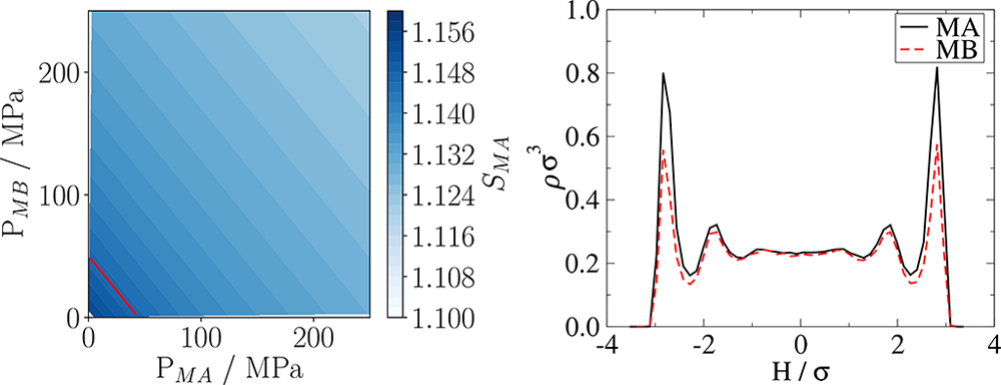
(Left) Pore selectivity as a function of partial pressures of MA and MB, with the red line (eq 20) being the region where transport diffusivities have been measured in this work. (right) Adsorbed densities of MA (solid black line) and MB (dashed red line) measured against pore height, for a system at equilibrium with a bulk equimolar mixture where ρ_MA_^Bulk^ σ_MA_^3^ = ρ_MB_^Bulk^ σ_MB_^3^ = 0.25.

Figure 11 (left) showcases pore selectivity, with selectivities of up to 16% at the lowest pressures (or bulk densities). With increasing pressure the pore become less selective. The main difference between the adsorption behavior of the two species is the amount adsorbed nearest to the pore surface, where species MA is adsorbed considerably more strongly than species MB, given the less favorable interaction between species MB and the solid. Free energy calculations found in the Supporting Information further showcase the stronger adsorption of species MA. With increasing densities, the first few monolayers near the pore surface become filled, and adsorption occurs in the middle of the pore, which is weakly affected by the solid surface, thus impartially allowing both species to adsorb in the middle resulting in a lower overall selectivity.

The region outlined by the red line in Figure 11 (left) was chosen as the subspace to measure the transport diffusivity matrix of the mixture. This region follows the following constraint:
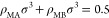
20where σ = σ_MA_ = σ_MB_.

In this subspace, the total bulk pressure fluctuates between 46.5 and 48.0 MPa. As the species adsorb differently inside the pore, by keeping the global composition constant, there are different amounts of MA and MB in the bulk at different compositions, leading to a slight difference in pressure. The composition defined using the mole fraction of species MA, *x*_MA_, is the independent variable. The pore selectivity remains constant independent of fluid composition, having a value of *S*_MA_ = 1.145.

**Figure 12:**
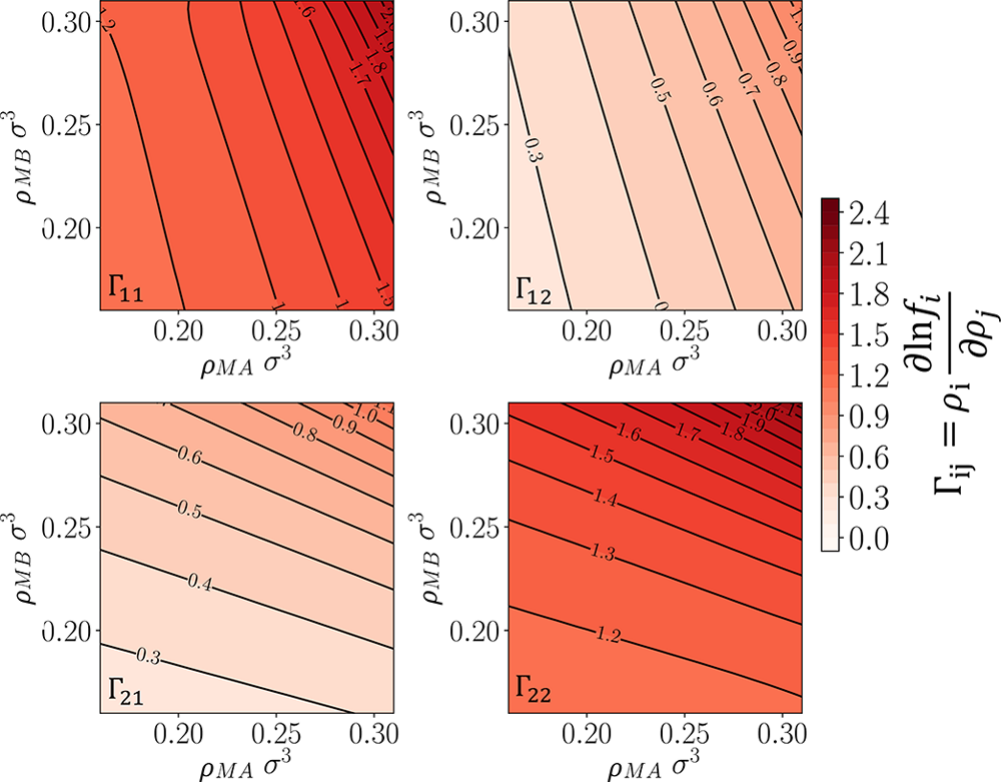
Darken factors of the binary methane-like fluids, where “1” refers to species MA and “2” refers to species MB.

**Figure 13:**
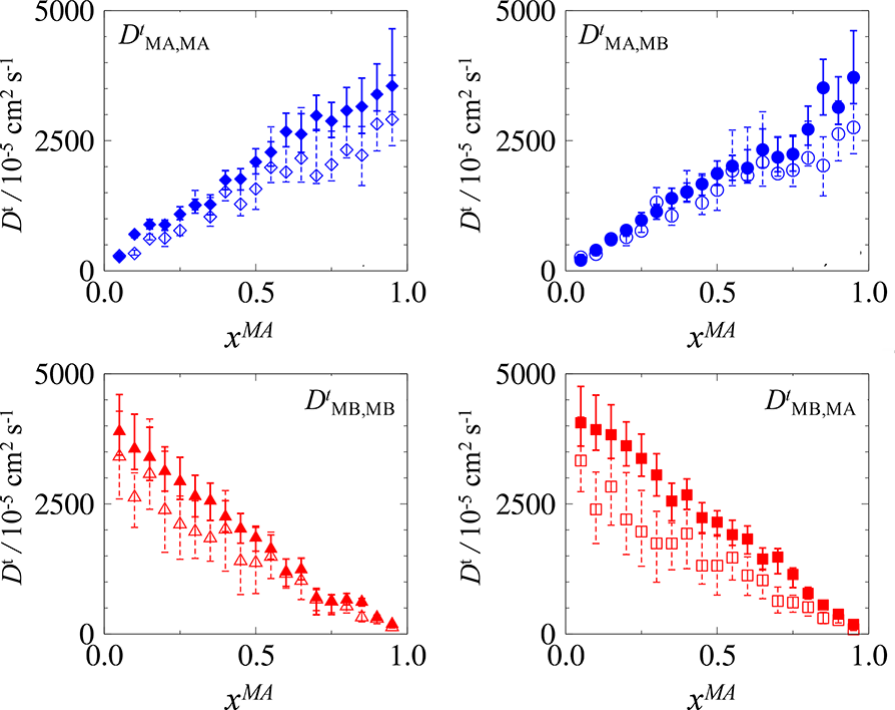
Transport diffusivities evaluated for the binary methane-like mixture at total reduced density of ∑*_i_*ρ_*i*_σ_*i*_^3^ = 0.5. Empty symbols are from the EqMD method, and filled symbols are from the BD-NEMD method.

To measure transport diffusivities from the EqMD method as benchmark cases, Darken factors were calculated using ∂ ln *f*/*∂ρ* as described in eq 13 (Figure 12). Generally, the values of the self-species Darken factor, Γ_*ii*_, increases with increasing concentrations of species *i*. Moreover, Γ_*ii*_ is much larger that the cross-species Darken factor, Γ_*ij*_, indicating that the fugacity of one species is not as strongly correlated with the changes of composition of the other species as it is with changes of compositions of itself. The transport diffusivity matrix is evaluated using both the EqMD and BD-NEMD methods. For the binary mixture, there are four Λ_*ij*_ and *D*_*ij*_^*t*^ values to be evaluated with eq 2 used to measure collective diffusivity matrix, [Λ], from the EqMD method. The matrix, along with Γ_*ij*_, is used to calculate the transport diffusivity matrix, [*D*^*t*^] using eq 13. The values of Λ_*ij*_ at different compositions can be found in the Supporting Information. In general, for this particular system, given the identical interactions between all fluid particles independent of species, it is found that Λ_MA,MA_ ≈ Λ_MA,MB_ and Λ_MB,MB_ ≈ Λ_MB,MA_, i.e., the flux of each species is equally influenced by chemical potential gradients of either species. This trend is also true for the transport diffusivities measured, and these can be seen in Figure 13, where filled symbols are transport diffusivities measured using the BD-NEMD method, and the empty symbols are those measured from the EqMD method. The first column describes the self-transport diffusivities, and the second column shows the cross coefficients, i.e., the contribution to the flux of species *i* due to concentrations gradient of species *j*.

*D*_*ii*_^*t*^ and *D*_*ij*_^*t*^ demonstrate positive linear correlations with molar composition of species *i*, with the diffusivity of each species approaching zero in the limit of zero concentration. For species MA, in the limit of pure component, i.e., *x*_MA_ → 1, *D*_MA,MA_^*t*^ approaches the value for the pure component system at ρσ^3^ = 0.5, presented in Figure 10. For Case II, *D*_BD-NEMD_^*t*^ ≈ 3800 × 10^–5^ cm^2^ s^–1^ which is in quantitative agreement in the limit of pure component for *D*_MA,MA_^*t*^. This is not the same for species MB, and the slope describing *D*_MB,MB_^*t*^ as a function of composition is steeper than that describing *D*_MA,MA_^*t*^. In the limit of pure component of each species, *D*_MB,MB_^*t*^ is 10% larger than *D*_MA,MA_^*t*^. This does not necessarily mean that species MB travels faster than species MA inside the pore, as a concentration gradient in each species causes significant fluxes in the other species. Nevertheless, the high transport coefficients of MB can be explained by the lower selectivity of species MB, as MA particles adsorb more strongly to the surface and are slightly slowed down. If we were interested in evaluating transport selectivity, then transport diffusivities could be used in continuum models to assess changes in composition downstream of a pore.

Commensurate with previous cases, it is seen that transport diffusivities measured using equilibrium methods have large uncertainties. Moreover, the boundary driven method consistently overestimates the diffusivity, with an average of 18% for all elements at all compositions. However, the trends are qualitatively consistent with the values measured using the BD-NEMD method within the uncertainty of the EqMD measurements. This deviation is not significant given that values of transport diffusivities span orders of magnitude with changing concentrations, as previously discussed for Cases I and II.

### Case IV: Binary Mixture of LJ Fluid with Size Difference

To understand the effect of size heterogeneity on the elements of the transport diffusivity matrix for a binary mixture, the system studied in Case I was modified by adding another species which is 30% larger than the original LJ fluid, keeping all other self-interaction parameters the same. The pore height is kept the same and the wall particles are thermostated at a temperature of 1.5 ε, resulting in a supercritical fluid. Henceforth the original LJ species will be referred to LJF-1, and the larger species will be referred to as LJF-2. From Table 1, it can be seen that the cross interaction energy, ε_*ij*_, between LJF-2 and LJF-1 and between LJF-2 and the solid (LJW) is slightly less than 200 K. This is a consequence of the combining rules used, where the cross interaction energy scales down if the two species have large size differences.

**Figure 14:**
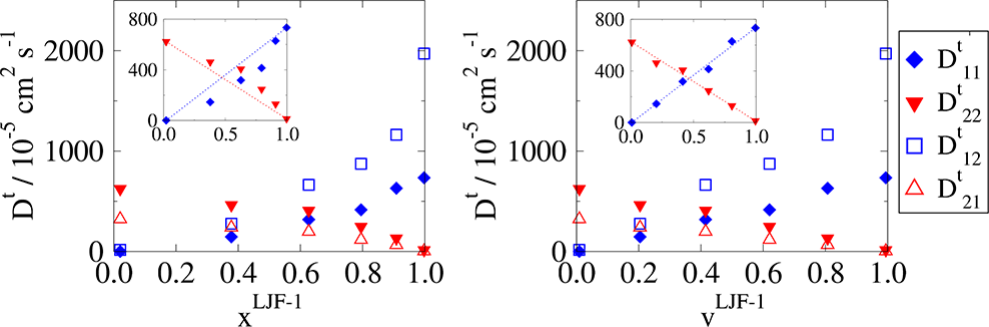
Transport diffusivities, *D*^*t*^, evaluated for Case IV LJ mixture at ∑_*i*_ρ_*i*_^Ads^σ_*i*_^3^ = 0.5. Here “1” and “2” refer to species LJF-1 and LJF-2 respectively. For this plot, total reduced density was maintained at ∑_*i*_ρ_*i*_σ_*i*_^3^=0.5. (left) Transport diffusivities measured against mole fraction of species LJF-1, *x*^LJF-1^. (right) *D*^*t*^ measured against volume fraction of said species, *v*^LJF-1^. The inset plots show the nonlinear relationship of *D*_*ii*_^*t*^ vs mole fraction and a linear relationship with volume fraction.

For this system, transport diffusivities were measured for all compositions where ∑_*i*_ρ_*i*_^Ads^σ_*i*_^3^ = 0.2–0.7. Compared with Case III, there are slight differences in the transport behavior in this system. To highlight the differences, the concentration constraint used to study Case III, i.e., ∑_*i*_ρ_*i*_^Ads^σ_*i*_^3^ = 0.5, was also investigated. The results can be seen in Figure 14 (left), where it can be seen that the relationship between transport diffusivity and mole fractions is no longer linear. At intermediate mole fractions, *x*^LJF-1^ ≈ 0.5, the self- and cross-transport diffusivities of species LJF-1 are lower than a linear correlation (*y* = *x*), and the self- and cross-transport coefficients of LJF-2 are conversely higher than expected. This is in agreement with previous studies, where strong correlation effects lead to the slowing down of a more mobile species and less strongly adsorbed species by the less mobile species.^31^ Without a linear relationship between mole fractions and transport coefficients, pure component transport diffusivities cannot be used to approximate the self-transport coefficients in the binary diffusivity matrix as functions of composition.

Interestingly, a linear relationship becomes apparent when transport is measured against volume fractions, *v* or by multiplying densities by σ^3^. When volume fraction of species *i*, *v*_*i*_, is zero, the self-transport diffusivity of species *D*_*ii*_^*t*^ is also zero. The self-transport coefficient linearly increases with increasing volume fractions until the point where *v*_*i*_ approaches 1, when its value approaches the pure component transport diffusivity.

As with previous cases, in this binary mixture, self-transport diffusivities are orders of magnitude larger than self-diffusivities. For the systems presented where reduced density is kept constant, self-diffusivities are independent of composition, having a value of 13 × 10^–5^ cm^2^ s^–1^ for LJF-1 and 10 × 10^–5^ cm^2^ s^–1^ for LJF-2. Again, this is very different to the transport diffusivities seen in Figure 14, emphasizing the fact that self-diffusivities are not adequate parameters to be used in understanding transport in mesoporous materials.

Moreover, for this system the cross coefficients behave differently from the ones studied in Case III. The cross transport coefficient for each species is not similar to the self-term. For species LJF-1, the cross term *D*_LJF-1,LJF-2_^*t*^ is larger than the self-term, *D*_LJF-1,LJF-1_^*t*^. For the other species, the opposite trend is observed. As previously described, the mutual diffusivity, *D*_*ji*_^*t*^, quantifies the influence of concentration gradients of species *i* on the flux of species *j*. To understand how the concentration gradient of species *i* affects the flow of both species (*i* and *j*), the ratio *D*_*ii*_^*t*^/*D*_*ji*_^*t*^ was compared with *x*_*i*_/*x*_*j*_ at different compositions. If the values of the diffusivity ratio are the same as the composition ratio, then it can be concluded that the flow is fully mutualized and that there is no transport selectivity. This is because each column of the transport diffusivity matrix describes the resultant fluxes emerging from the same concentration gradient, and if the ratio of the elements of each column of the transport diffusivity matrix ratio is the same as the ratio of molar compositions in the pore, then it implies that the fluid is fully mixed, i.e., “toothpaste” or ideal piston flow. An exhaustive discussion is provided in the Supporting Information. Table 2 shows the comparison of ratios and a quantitative agreement is observed.

**Table 2:** Ratio of the Elements in Each Column of the Transport Diffusivity Matrix^a^

*v*_2_ (%)	*D*_1,2_^*t*^/*D*_2,2_^*t*^	*D*_1,1_^*t*^/*D*_2,1_^*t*^	*x*_1_/*x*_2_
20	1.644	1.672	1.682
40	0.622	0.613	0.638
60	0.283	0.282	0.265
80	0.105	0.112	0.095

a The results agree well with the ratio of the molar compositions at different volume fractions at ∑_*i*_ρ_*i*_^Ads^σ_*i*_^3^ = 0.5. This agreement alludes to the fact that the flow is fully mutualized. The subscripts “1” and “2” refer to species LJF-1 and LJF-2 respectively.

Although the transport is fully mutualized, composition still affects the overall transport. Increasing the composition of the lighter species leads to a faster flow, which can be quantified by measuring the total fluxes and the total density gradient across the pore (refer to the Supporting Information). The smaller species acts as a diluting agent for the larger species, enhancing overall transport.

In this case, the self-transport coefficient of the smaller species is at least 20% larger than that of the larger one in the pure component limit, and the cross coefficient of the smaller species is 7 times larger. Although these metrics would ostensibly allude to a faster transport of species LJF-1, this is not the correct conclusion, as the fluid is fully mutualized, yet it could be seen that for high density fluids inside slit pores, if the adsorption is ideal and the fluid is well-mixed, then the self-transport diffusivity of each component can be estimated using the linear relationship with respect to the volume fractions. Moreover, if the transport is fully mutualized, then the cross terms can be estimated using the ratio of the molar compositions.

The complete picture of transport diffusivity matrix of this system as a function of the adsorbed concentration can be found in the Supporting Information.

## Conclusions

This work was carried out to validate the assumptions currently used in the BD-NEMD methods in measuring transport diffusivity of pure components. By benchmarking values of transport diffusivity obtained using the BD-NEMD method against those from the EqMD method, it was highlighted that current implementations of this nonequilibrium method, where it is assumed that concentration gradients can be calculated using bulk reservoir concentrations on either side of the pore, lead to spurious values of transport diffusivity. By relating bulk concentrations on each side to their adsorbed concentrations at equilibrium and using adsorbed concentration gradients, it was shown that the BD-NEMD method can be used to compute accurately transport diffusivities with much lower uncertainty than the EqMD method.

To relate EqMD collective diffusivities to transport diffusivities, Darken correction factors are required. Commonly, these correction factors are assessed using GCMC methods. In this work, a novel method was introduced, whereby the Darken factors are evaluated for systems where intermolecular interactions are described using Mie potentials. Using the same system used for the BD-NEMD method at zero force, the equilibrium bulk compositions for each system are used to calculate chemical potentials from the molecular based equation of state, SAFT-γ Mie. A Python code for the evaluation of the SAFT-γ Mie EoS is available.^105^

For dense fluids and liquids, it is shown that transport coefficients are orders of magnitude larger than self-diffusivities. Although there is no formal expectation that these two quantities match except at the ideal gas limit, it is a frequent working assumption which is proven wrong.

In dense binary systems, the influence of the adsorption selectivity on transport is minimal, at least for modest differences in pore attraction. The influence of the size of the particles is, on the contrary, more pronounced. Larger particles dominate the transport, and it is seen that the transport cross coefficients become relevant. These significant values of cross coefficients result in mutualized flow in slit pores at high densities, i.e., no transport selectivity is observed. We do make the caveat that these heuristic observations are relevant only to smooth pores, and a significant difference is seen upon the consideration of transport in rugous nanopores, as we will describe in a future communication.

## Supporting Information Available

Full details of the molecular interactions; pore structure and pore height characterization; thermostating; accuracy of the methane model in describing thermodynamic and transport properties; ideal adsorption solution theory for binary mixtures; density profiles inside the pores; enhanced discussion on flow mutualization and transport inside the pores; binary transport coefficients for cases III and IV; free energy calculations of binary mixtures (PDF)
