# Supplementary material for: Use of Boundary-Driven Nonequilibrium Molecular Dynamics for Determining Transport Diffusivities of Multicomponent Mixtures in Nanoporous Materials
Source: J Phys Chem B. 2022 Feb 1;126(5):1085–100. doi: 10.1021/acs.jpcb.1c09159 (PMC9007456; doi:10.1021/acs.jpcb.1c09159)
Supplement: Supplementary file 1 — jp1c09159_si_001.pdf [file jp1c09159_si_001.pdf]

Supporting Information: On the Use of Boundary Driven Non-Equilibrium Molecular Dynamics for Determining Transport Diffusivities of Multicomponent Mixtures in Nanoporous Materials

Maziar Fayaz-Torshizi,[†] Weilun Xu,[†] Joseph R. Vella,[‡,§] Bennett D. Marshall,[¶] Peter I. Ravikovitch,[¶] and Erich A. Müller[*,†]

†*Department of Chemical Engineering, Imperial College London, UK*

‡*ExxonMobil Research and Engineering Company, TX, USA*

¶*ExxonMobil Research and Engineering Company, Annandale, NJ, USA*

§*Current Address: Princeton Plasma Physics Laboratory (PPPL), NJ, USA*

E-mail: e.muller@imperial.ac.uk

Molecular Interactions

All fluid molecules are modelled as single spheres, with the Mie potential describing intermolecular interactions between particles i and j:

$$u_{ij}^{Mie}(r) = \mathcal{C}\varepsilon_{ij}\left[\left(\frac{\sigma_{ij}}{r}\right)^{\lambda_{ij}} - \left(\frac{\sigma_{ij}}{r}\right)^{6}\right] \tag{1}$$

where ε_{ij} is the depth of the potential well, σ_{ij} is the average distance between the centres of mass of the two segments at contact, and λ_{ij} is the repulsive exponent, controlling the softness and the range of the interaction between two segments. $\mathcal{C}$ is a pre-factor:

$$\mathcal{C} = \left(\frac{\lambda_{ij}}{\lambda_{ij}-6}\right)\left(\frac{\lambda_{ij}}{6}\right)^{6/(\lambda_{ij}-6)} \tag{2}$$

For the case where λ_{ij} is equal to 12, the Mie potential is equivalent to the Lennard-Jones (LJ) potential. For multicomponent systems, the following combining rules are applied:

$$\begin{aligned} \sigma_{ij} &= \tfrac{1}{2}(\sigma_{ii} + \sigma_{jj}) \\ (\lambda_{ij} - 3) &= \sqrt{(\lambda_{ii} - 3)(\lambda_{jj} - 3)} \\ \varepsilon_{ij} &= \tfrac{(\sigma_{ii}^3 \ \sigma_{jj}^3)^{1/2}}{\sigma_{ij}^3}\sqrt{\varepsilon_{ii} \ \varepsilon_{jj}} \end{aligned} \tag{3}$$

Where ii and jj refer to the pure component (self) parameters.

Pore Structure and Pore Height Definition

Pores are modelled as mesoporous materials, i.e. smooth slit pores, composed of solid particles in a face centred cubic (FCC) lattice. Inside the pore, the fluid is in contact with the (111) plane of the lattice, where the particles in the first layer closest to the fluid form hexagonal patterns. Conclusions made about transport in these materials is therefore only strictly related to smooth slit pores.

In order to clearly define the adsorbed densities and fluxes, it is important to address

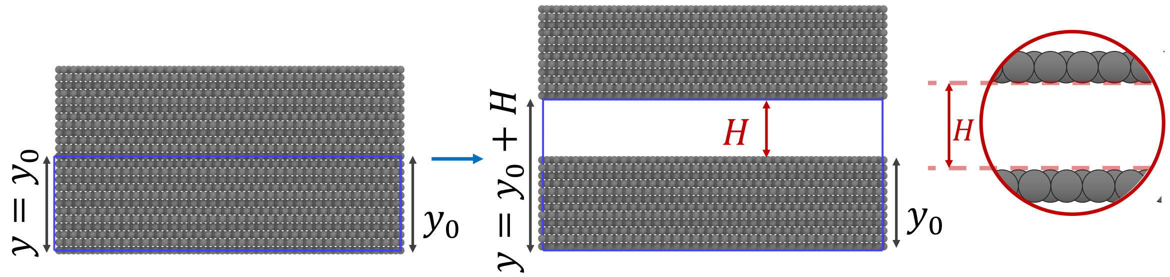

Figure S1: Pore height is systematically defined by having a fully periodic FCC solid with no accessible pore height, and then increasing the y dimension of the simulation box by the value of the pore height, H.

the issue of how one defines the pore height. The definition of the pore height can be best understood using Figure S1. First, a packed solid structure is generated, fully periodic in all dimensions. The y dimension of the simulation box is increased by the pore height. The height, H, corresponds to the distance between the planes defined by the outer most points of the exposed surface beads defined by their characteristic diameter. Clearly, other definitions of H may be used.[1,2] Alternative definitions of it will not have influence on the main conclusions of this work.

All walls are modelled as FCC lattices, and for all cases, the lattice constant is $a = 2\sqrt{2}\,\sigma^{Wall}$.

Ideal adsorption solution theory(IAST)

Single component adsorption isotherms can be readily measured using simulations and experiments,[3] however it is more challenging to map out the adsorption isotherms of a multi-component fluid because knowledge on fluid compositions are required to get the full picture.[4].[5] To enable efficient estimations of mixture adsorption, Myers and Prausnitz developed ideal adsorption solution theory (IAST), which is now a widely used tool to estimate mixture adsorbed isotherm from the single-component isotherms of the constitute fluids.[6,7] The theory is based on the assumption that the adsorbed fluid is an ideal solution in which interaction between fluid molecules are equivalent in strength.[8] Because this ideal adsorbed phase is equilibrated with a bulk phase, IAST can be seen as an analogy of Raoult's law which computes the adsorbed phase using bulk pressure and composition.[9] Methods for implementing IAST have been extensively discussed since its emergence and details can be found in literature.[10]

The spreading pressure of absorbed pure component, i, in equilibrium with a its pure component gas at pressure P_i° can be calculated using

$$\frac{\pi_i A}{RT} = \int_0^{P_i^\circ} \frac{n_i(P_i^o)}{P_i} dP_i \tag{4}$$

Where π_i is the spreading pressure, A is the total area available on the adsorbents, assumed to be the same for all adsorbates. n_i is the moles of the adsorbed component at pressure P_i^o, with is given by the pure-component adsorption isotherm. IAST states that equilibrium is reached between adsorbed phase and bulk phase, therefore the spreading pressure for all fluids for a given isotherms should be equal:

$$\pi = \pi_i(P_i^\circ) = \pi_j(P_j^\circ) = ... = \pi_N(P_N^\circ) \tag{5}$$

For an adsorbable component, i, at equilibrium, the analogous Raoult's law is:

$$P_i = y_i P = x_i P_i^o \tag{6}$$

Where P_i is the partial pressure of fluid i in bulk phase, P is the total bulk pressure, y_i is the molar compostion of i in bulk phase, x_i is the molar composition of fluid i in adsorbed phase, and P_i^o is the partial pressure of pure fluid i which yields the same spreading pressure π as the mixture. For a given multi-component adsorption isotherm with known bulk pressure and composition, one can combine Equations (5) and (6) to compute the adsorbed phase composition x_i. The total adsorbed amount N_T can then be evaluated using the following equation

$$\frac{1}{N_T} = \sum_{i=1}^{N} \frac{x_i}{n_i^\circ P_i^\circ} \tag{7}$$

Where n_T is the total mole of adsorbed phase, n_i^o is the adsorbed fluid of pure fluid i derived from pure adsorption isotherm. In this paper, the IAST method is implemented using the pyIAST package by Haxranczyk et al.[11]

Thermostating

As mentioned in the paper, in order to minimise external influences on the dynamics of the fluid, during the production of the NEMD and EqMD simulations, thermostating was only applied to the wall particles. However, this doesn't affect the temperature of the fluid as the fluid is in contact with the wall and the wall particles add of remove energy to the fluid so that the temperature of the fluid remains the same as the wall temperature. An example is shown for for the Case IV, where NEMD simulations are run and the highest force used in this work is applied to both species. As can be seen, the temperature coupling method is robust in controlling the temperature of the fluid.

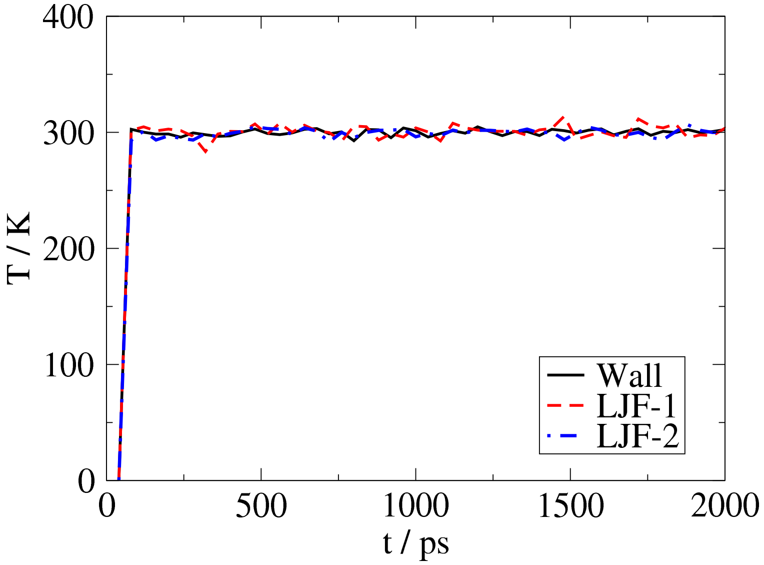

Figure S2: Applying thermostating to the walls keeps the fluid temperature controlled: an example of Case IV where reduce density is kept at 50 % and $x_{LJF-1} = 0.5$. An acceleration of 0.004 nm ps^{-2} is applied to both species.

Case I : Pure LJ density profile

In order to reach a specific global density, two copies of bulk region are set up and connected to an empty pore. The two bulk regions act as reservoir therefore the desired global density can be reached by manipulating the density in the bulk. Simulation were run in the NVT ensemble so that the fluid reach equilibrium in the pore. Figure S3 shows the adsorbed density profile across a range of global densities.

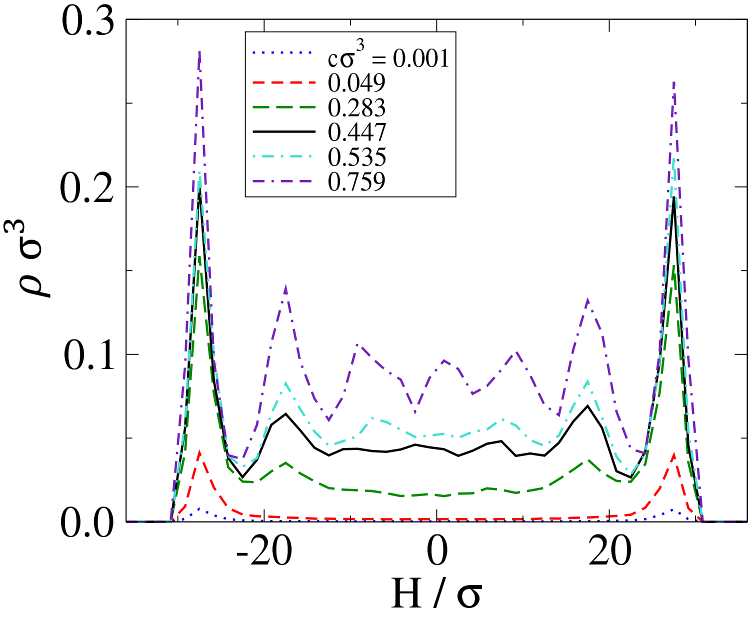

Figure S3: Density profiles inside the pore, where H refers to the height of the pore. Legend corresponds to global densities.

Case I: Relationship between chemical potential, μ, and adsorbed density, ρ^{Ads}

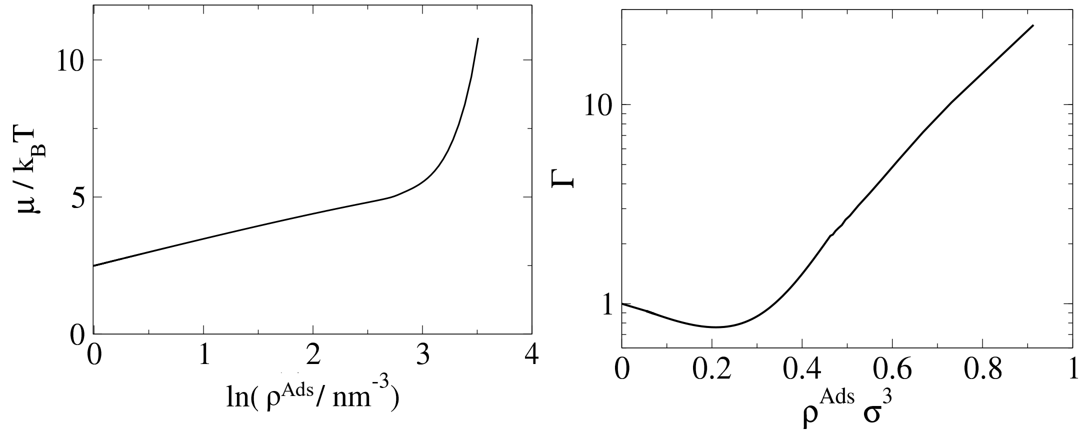

Figure S4: ***Left***: Chemical potential vs $\ln(\rho^{Ads})$ from SAFT-γ Mie EoS. The slope of this curve is the Darken factor. ***Right***: The Darken factor, Γ, vs ρ^{ads} calculated from the left plot.

Case II: Thermodynamic and transport properties of the methane model used in this work

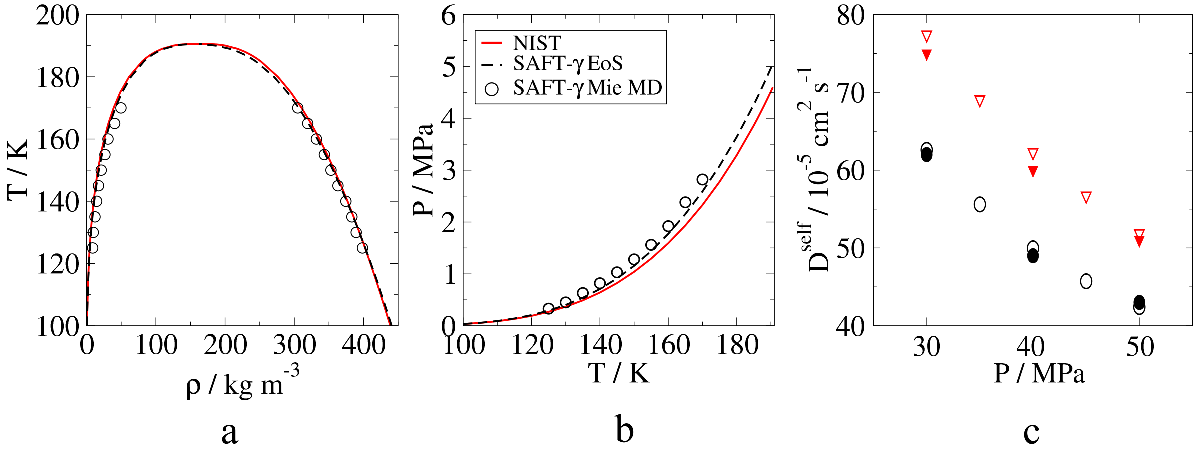

Figure S5: Comparison between experimental data and the SAFT-γ EoS and MD simulations describing saturation properties of methane. ***a*** temperature vs saturated densities, ***b*** vapour pressures vs temperature. Red line corresponds to smoothed experimental data,[12] open symbols are simulations while dashed lines are the results of the equation of state, the latter two employing the same molecular parameters. ***c*** Self diffusivity of methane predicted using MD simulations in this work (empty symbols) as compared with literature (filled symbols),[13] triangles and circles correspond to T = 303 K, and T = 333 K respectively.

Case II: Adsorbed isotherms, adsorbed density profile and chemical potential

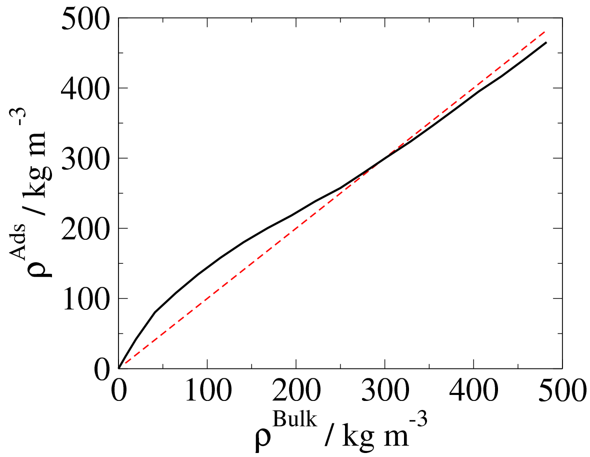

Figure S6: Adsorption isotherm of methane at 298K, averaged from 100 million time steps.

The adsorption isotherm of methane can be found in figure S6. The red dotted line refers to $\rho_{Bulk} = \rho_{Ads}$, which highlights the strong adsorption effect when bulk density is below 250 Kg/m^3. However, the adsorption effect becomes less noticeable at higher pore loading, evidenced by the fact that the adsorption isotherm approaches the reference line as bulk density increases. Figure S7 presents the adsorbed methane density profile across a range of global density. Figure S8 shows the SAFT-generated methane chemical potential at different densities. The slope of this plot is further used to evaluate the Darken factor at any given methane density.

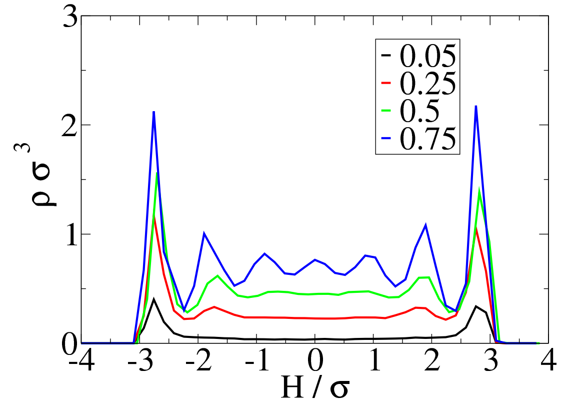

Figure S7: Examples of adsorbed methane density profile in slit pore at a range of global density. Legend refers to reduced densities.

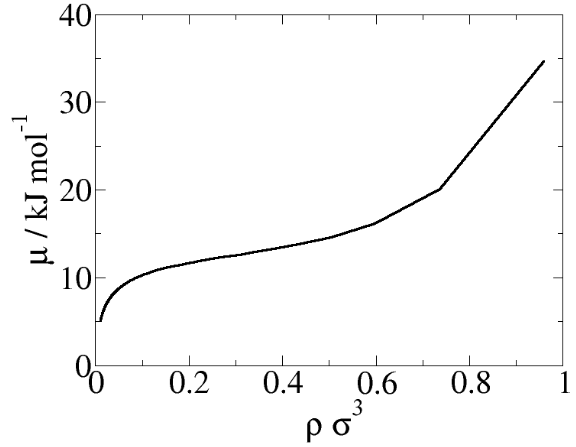

Figure S8: SAFT generated chemical potential of bulk methane at a range of densities, the slope of this plot gives the local Darken factor

Case III: Pure Component Adsorption Isotherms and Binary Adsorption using IAST

Adsorption isotherm of the binary mixture is presented in Figure S9.

In order to validate the IAST theory on MA/MB mixture, systems with constant global density but different compositions are set up and equilibrated under NVT ensemble. Bulk pressure and bulk composition were then measured to estimate their corresponding pore density using IAST. This IAST estimation is then compared to the pore density measured

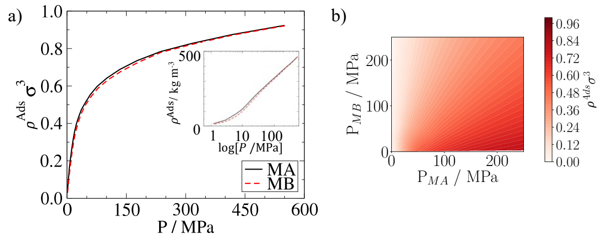

Figure S9: ***a***: Pure component adsorption isotherms of species MA (solid line) and MB (dashed line) at T = 298 K. ***b***: Adsorption isotherm for species MA as a function of partial pressures of MA and MB.

directly from the simulation. Figure S10 shows such comparison at 0.5 reduced global density (same density studied in case III), which prove that IAST gives excellent predictions on the adsorption of MA/MB mixture.

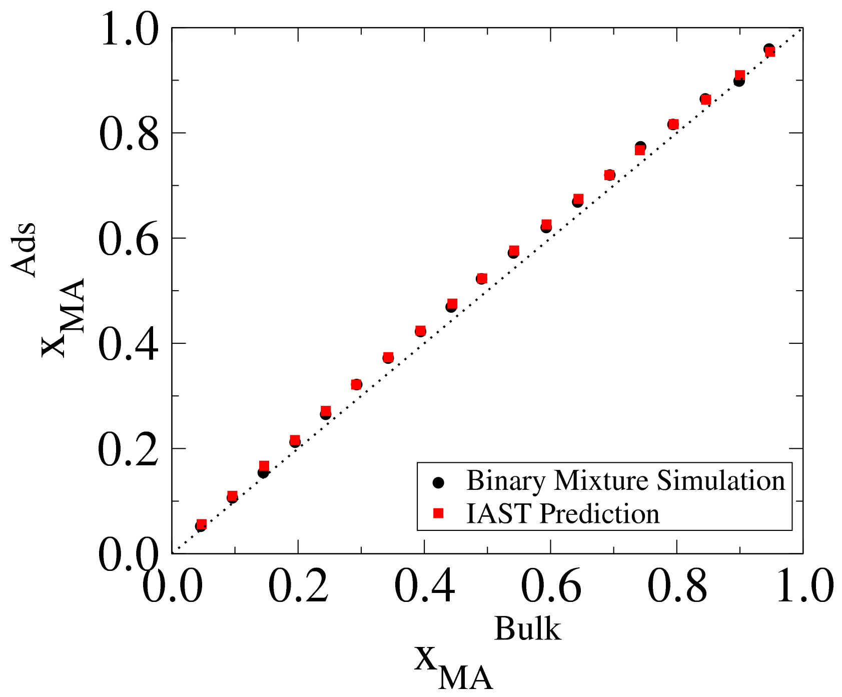

Figure S10: Prediction of the adsorption of the binary mixture of studied in Case III at different compositions for a fixed mixture global density, $\sum_i \rho_i \sigma_i^3 = 0.5$, corresponding to a pressure 46 MPa. IAST predicts binary adsorption using pure component adsorption isotherms. Both IAST and binary mixture simulations indicate a slight selectivity towards species MA, which has a higher fluid solid interaction than species MB.

Case III: Free energy calculation

Free energy calculation of MA/MB mixtures shows that MA has a higher energy level inside the pore, proving that MA adsorbs stronger than MB.

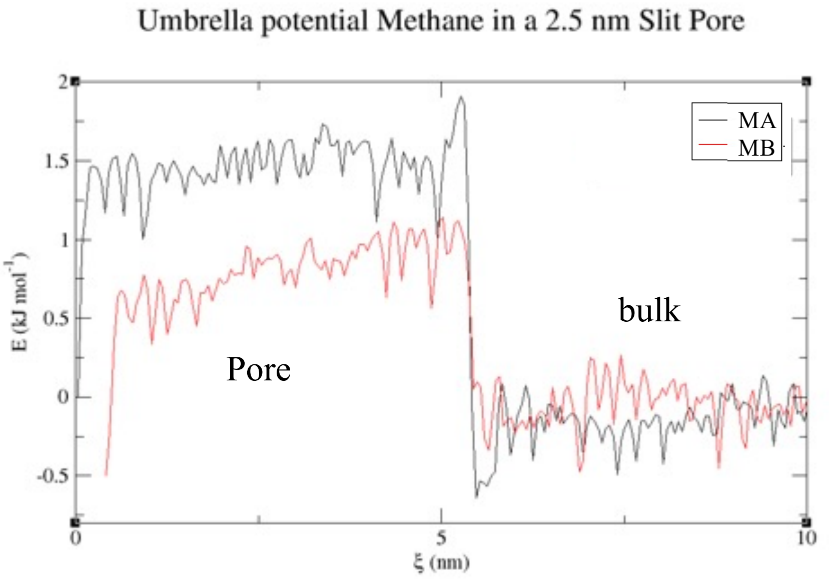

Figure S11: Free energy calculation of MA/MB mixtures in 2.5nm slit pore.

Case III: Λ_{ij} values

EqMD were run on a range of composition of MA/MB mixture at 0.5 total reduced density, Figure S12 shows the collective diffusivity Λ_{ij} resulted from those simulations. From this, the transport diffusivity D^t_{ij} were then calculated using collective diffusivity Λ_{ij} and the local Darken factor Γ_{ij}.

$$D^t_{ij} = \sum_{k=1}^{\#\ Comps} \beta\rho_k \frac{\partial \mu_k}{\partial \rho_j} \Lambda_{ik} = \sum_{k=1}^{\#\ Comps} \rho_k \frac{\partial \ln f_k}{\partial \rho_j} \Lambda_{ik} = \sum_{k=1}^{\#\ Comps} \Gamma_{kj}\Lambda_{ik} \tag{8}$$

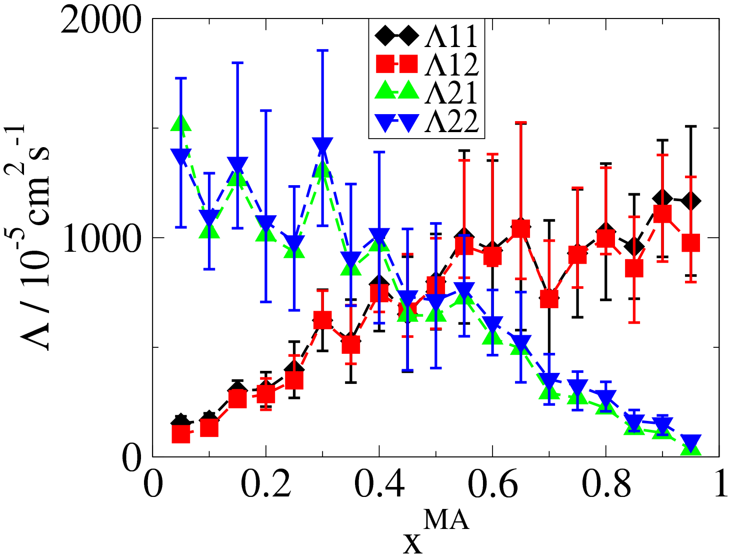

Figure S12: Collective diffusivity matrix Λ_{ij} evlauted from EqMD of MA/MB binary mixture at 0.5 total reduced density. Component number 1 and 2 represents MA and MB respectively

Case IV: Discussion on transport selectivity

One way to confirm mutualisation in slit pores is by observing velocity profiles from external-force (EF-NEMD) simulations,[14] when the force is only applied to one species. This is explored in Figure S13 (Left), where it can be seen that by applying forces to all particles of species LJF-2, the velocities of species LJF-1 and LJF -2 are the same, confirming the high correlation between the movement of all molecules.

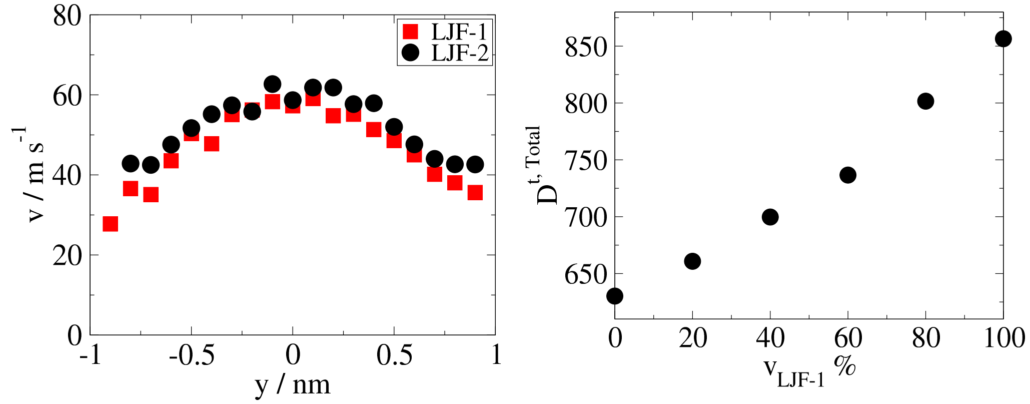

Figure S13: ***Left*** Velocity profiles of species LJF-1 (red squares) and LJF-2 (black circles) when a directional acceleration (0.01 nm ps^{-2}) is applied to all particles of species LJF-2, where $v_{LJF-2} = 0.4$ and $\sum_i \rho_i^{Ads} \sigma_i^3 = 0.5$. ***Right*** Transport diffusivity of the entire fluid, $D^{t,Total}$ vs fluid volume fraction. Increasing the concentration of the lighter species increases the overall transport of the fluid.

In order to assess this using transport coefficients, we could refer to the theory and the values of transport coefficients.

For a binary fluid, it is understood that the transport of each component is affected by driving forces originating from both components. We can write this correlation as a matrix equation

$$\begin{aligned} J_1 &= D_{11}^t \nabla \rho_1 + D_{12}^t \nabla \rho_2 \\ J_2 &= D_{21}^t \nabla \rho_1 + D_{22}^t \nabla \rho_2 \end{aligned} \tag{9}$$

Where D_{ij}^t is the mutual diffusivity which quantifies the influence of concentration gradients of species i on the flux of species j.

In order to evaluate transport selectivity, one must first calculate the ratio of the fluxes

between two species:

$$R^{flux} = \frac{J_1}{J_2} = \frac{D^t_{11}\nabla\rho_1 + D^t_{12}\nabla\rho_2}{D^t_{21}\nabla\rho_1 + D^t_{22}\nabla\rho_2} \tag{10}$$

If the ratio of the fluxes is different to the ratio of the molar compositions, i.e. x_1/x_2, then there is an observed transport selectivity, as it suggests that the amount of one species flowing is faster than the other species, leading to a different molar flux. Thus transport selectivity can be defined as:

$$S^{Trans} = \frac{J_1}{J_2}/\frac{x_1}{x_2} = \Big(\frac{D^t_{11}\nabla\rho_1 + D^t_{12}\nabla\rho_2}{D^t_{21}\nabla\rho_1 + D^t_{22}\nabla\rho_2}\Big)\frac{x_2}{x_1} \tag{11}$$

Independent of the values of $\nabla\rho_1$ and $\nabla\rho_2$, S^{Trans} can equal one if:

$$D^t_{11}/D^t_{21} = x_1/x_2 = D^t_{12}/D^t_{22} = x_1/x_2 \tag{12}$$

Case IV: binary transport diffusivity coefficients

Transport diffusivity matrix of the LJ binary system with respect to the change of density of both fluids.

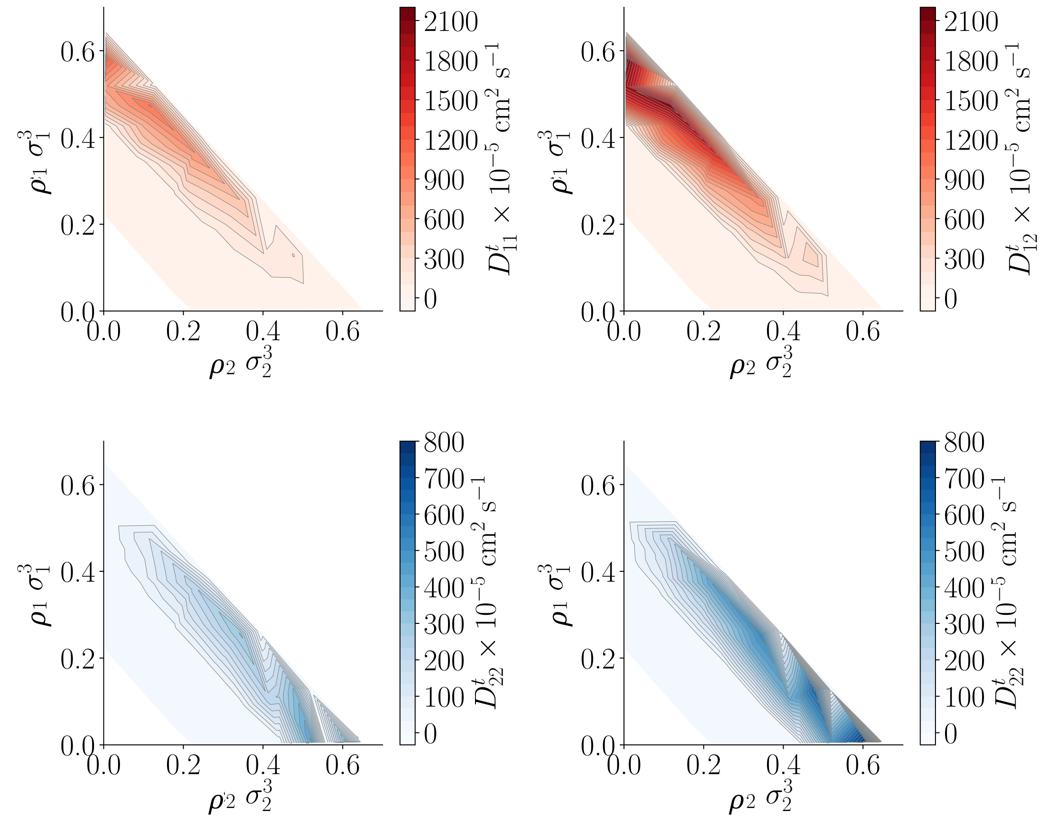

Figure S14: Elements of the binary transport diffusivity coefficients of the binary fluid for Case IV. Like previous figures, 1 and 2 refer to species LJF-1 and LJF-2 respectively.

References

(1) Kaneko, K. Determination of pore size and pore size distribution: 1. Adsorbents and catalysts. *Journal of membrane science* **1994**, *96*, 59–89.

(2) Herrero, C.; Omori, T.; Yamaguchi, Y.; Joly, L. Shear force measurement of the hydrodynamic wall position in molecular dynamics. *The Journal of chemical physics* **2019**, *151*, 041103.

(3) Mason, J. A.; McDonald, T.; Bae, T.; Bachman, J.; Sumida, K.; Dutton, J.; Kaye, S.; Long, J. Application of a High-Throughput Analyzer in Evaluating Solid Adsorbents for Post-Combustion Carbon Capture via Multicomponent Adsorption of CO2, N2, and H2O. *J.Am.Chem.Soc.* **2015**, *134*, 4787–4803.

(4) Talu, O. Measurement and Analysis of Mixture Adsorption Equilibrium in Porous Solids. *Chem.Ing.Tech* **2011**, 67–82.

(5) Sircar, S. Basic Research Needs for Design of Adsorptive Gas Separation Processes. *Ind.Eng.Chem.Res* **2006**, *45*, 5435–5448.

(6) Myers.A.L, P. Thermodynamics of mixed-gas adsorption. *AlChE journal* **1965**, *11*, 121–127.

(7) Walton, K. S.; Sholl, D. S. Predicting multicomponent adsorption: 50 years of the ideal adsorbed solution theory. *AlChE journal* **2015**, *61*, 2757–2762.

(8) Bartholdy, S.; Bjorner, M.; Solbraa, E.; Shapiro, A.; Kontogeorgis, G. Capabilities and limitations of predictive engineering theories for multicomponent adsorption. *nd Eng Chem Res.* **2013**, *52*, 11552–11563.

(9) Yang, R. T. *Gas separation by adsorption processes*; Imperial College Press, 1988.

(10) Landa HOR, S. A., Flockerzi D A method for efficiently solving the iast equations with an application to adsorber dynamics. *AlChE journal* **2013**, *59*, 1263–1277.

(11) Simon, C. M.; Smit, B.; Haranczyk, M. pyIAST: Ideal adsorbed solution theory (IAST) Python package. *Computer Physics Communications* **2016**, *200*, 364–380.

(12) Kroenlein, K.; Muzny, C.; Kazakov, A.; Diky, V.; Chirico, R.; Magee, J.; Abdulagatov, I.; Frenkel, M. NIST Standard Reference 203: TRC Web Thermo Tables (WTT) Version 2-2012-1 Professional. *National Institute of Standards and Technology*

(13) Helbaek, M.; Hafskjold, B.; Dysthe, D. K.; Sørland, G. Self-Diffusion Coefficients of Methane or Ethane Mixtures with Hydrocarbons at High Pressure by NMR. *Journal of Chemical & Engineering Data* **2019**, *41*, 598–603.

(14) J Evans, D.; P Morriss, G. *Statistical mechanics of nonequilbrium liquids*; ANU Press, 2007.
